# Unconventional protein post-translational modifications: the helmsmen in breast cancer

**DOI:** 10.1186/s13578-022-00756-z

**Published:** 2022-02-25

**Authors:** Jiena Liu, Qin Wang, Yujuan Kang, Shouping Xu, Da Pang

**Affiliations:** 1 Department of Breast Surgery, Harbin Medical University Cancer Hospital, Harbin, 150040 China; 2 Department of Pharmacology (The State-Province Key Laboratories of Biomedicine-Pharmaceutics of China), College of Pharmacy of Harbin Medical University, Harbin, 150086 China; 3 Heilongjiang Academy of Medical Sciences, Harbin, 150086 China

**Keywords:** Breast cancer, Post-translational modifications, Oncogenesis, Proteomics

## Abstract

Breast cancer is the most prevalent malignant tumor and a leading cause of mortality among females worldwide. The tumorigenesis and progression of breast cancer involve complex pathophysiological processes, which may be mediated by post-translational modifications (PTMs) of proteins, stimulated by various genes and signaling pathways. Studies into PTMs have long been dominated by the investigation of protein phosphorylation and histone epigenetic modifications. However, with great advances in proteomic techniques, several other PTMs, such as acetylation, glycosylation, sumoylation, methylation, ubiquitination, citrullination, and palmitoylation have been confirmed in breast cancer. Nevertheless, the mechanisms, effects, and inhibitors of these unconventional PTMs (particularly, the non-histone modifications other than phosphorylation) received comparatively little attention. Therefore, in this review, we illustrate the functions of these PTMs and highlight their impact on the oncogenesis and progression of breast cancer. Identification of novel potential therapeutic drugs targeting PTMs and development of biological markers for the detection of breast cancer would be significantly valuable for the efficient selection of therapeutic regimens and prediction of disease prognosis in patients with breast cancer.

**Supplementary Information:**

The online version contains supplementary material available at 10.1186/s13578-022-00756-z.

## Introduction

Breast cancer is a heterogeneous disease with a high incidence and mortality rate among females worldwide [1, 2]. Although there have been significant advances in the conventional breast cancer treatment involving surgery, radiation, and chemotherapy, many patients develop resistance to these therapies during the course of disease progression, eventually resulting in cancer recurrence and metastasis [3]. Therefore, it is crucial to develop novel therapies for the treatment of breast cancer.

**Fig. 1:**
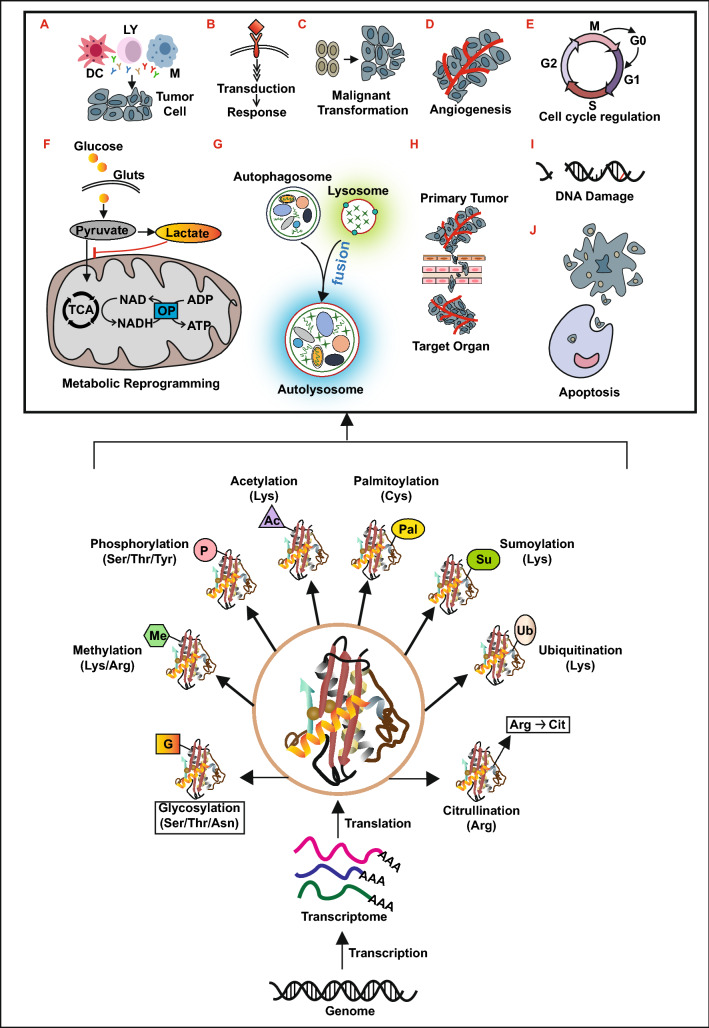
Overview of post-translational modifications in breast cancer. LY: Lymphocyte; M: macrophage; DC: dendritic cells; GLUTs: Glucose Transporters; OP: oxidative phosphorylation; TCA: tricarboxylic acid cycle; Ser: serine; Thr: threonine; Asn: asparagine; Lys: lysine; Arg: arginine; Tyr: tyrosine; Cys: cysteine; Cit: citrulline

Genetic code determines the specific sequence of a protein and whose function can be regulated by different modifications after translation. PTMs are defined as the chemical modifications of a protein that take place after its translation [4]. Protein PTMs increase the diversity of protein by altering their physical and chemical properties, conformation, and binding capacity. It is estimated that 50%-90% of proteins in human body undergo PTMs [5]. Each type of PTMs mainly consists of three components, writers, which add the modifications to the substrates; erasers, which wipe off the modification from the substrates; and readers, which recognize and bind the modified substrates to perform the corresponding biological functions. The writers, readers, and erasers are consisting of various enzymes [6–10]. The processes of PTMs are fine-tuned by thousands of enzymes and whose dysregulation contributes to a variety of pathologies that can be the primary driver of cancer [11–13]. Therefore, the study of PTMs is particularly valuable in cases where cancer cells do not differ in the expression or mutational status of a protein in the pathological process. Over the past few decades, due to significant advances in genomic, proteomic, bioinformatics, and mass spectrometric technologies, several enzymes (Additional file 1: Table S1) such as lysine acetyltransferases (KATs), deacetylases (KDACs), protein lysine methyltransferases (PKMTs), protein arginine methyltransferases (PRMTs) and so on that involved in the regulation of PTMs have been discovered in breast cancer [14–22]. Furthermore, several proteins (Additional file 1: Table S1) that undergone a series of PTMs, playing significant roles in the etiology of breast cancer, have been identified [14–16, 23]. These PTMs, including phosphorylation, ubiquitination, sumoylation, citrullination, acetylation, methylation, glycosylation, and palmitoylation (Fig. 1), take place in numerous proteins to regulate their stability, activity, cellular localization, interaction with other macromolecules, and the cellular response to different stimuli [9, 15, 24, 25]. PTMs have been shown to mediate several cellular pathophysiological processes related to breast cancer [24], such as immune response (Fig. 1A), signal transduction (Fig. 1B), cell proliferation, malignant transformation (Fig. 1C), angiogenesis (Fig. 1D), cell cycle regulation (Fig. 1E), metabolic reprogramming (Fig. 1F), autophagy (Fig. 1G), cell epithelial-mesenchymal transition (EMT) and invasion (Fig. 1H), as well as DNA damage repair (Fig. 1I) and apoptosis (Fig. 1J). Furthermore, several studies have reported that the dysregulation of PTMs plays a crucial role in the onset and progression of breast cancer [14, 19, 23, 26–29]. However, the exact molecular mechanisms underlying the effects of these abnormal PTMs on the various pathophysiological processes in breast cancer have not yet been extensively studied.

The target protein of PTMs can be classified into two categories, histones and non-histones. Histone modifications, especially the modifications that take place in H3 and H4, regulate the structure of chromatin to promote transcriptional activation by relaxing chromatin and induce transcriptional repression through condensing chromatin [30, 31]. Therefore, the modifications that occur on histones are also known as epigenetic modifications [30–32]. Protein phosphorylation is a sophisticated network consists of protein kinases, substrates, phospho-binding proteins and phosphatases [33–37]. During evolution, protein phosphorylation emerged as an essential and the most prevalent post-translational modification due to its variability and reversibility [33, 35, 38]. A breakthrough in the treatment of human epidermal growth factor receptor 2 (HER2) positive breast cancer patients was achieved by the discovery of tyrosine kinases (RTKs) inhibitors targeting protein phosphorylation, namely, trastuzumab and gefitinib [39–41]. Therefore, we wonder whether PTMs, other than protein phosphorylation, could also be potential therapeutic targets in breast cancer. In the context that histone epigenetic modifications and phosphorylation have been studied intensively in breast cancer [42–45]. However, it has become increasingly clear that other unconventional PTMs, such as acetylation, glycosylation, sumoylation, methylation, ubiquitination, citrullination, and palmitoylation, play equally important effects on the occurrence and progression of breast cancer [17, 18, 46–50]. So in this review, we introduce the relationship between these unconventional PTMs and breast cancer from the point of view of their underlying mechanisms in the oncogenesis and cancer progression. Moreover, we summarize the inhibitors targeting unconventional PTMs and the various PTM-associated clinical trials and thus, present the therapeutic potential of PTMs in breast cancer.

## Protein acetylation

### Potential mechanisms of protein acetylation involved in breast cancer

Protein acetylation is a reversible and evolutionarily conserved PTM regulated by the opposing actions of KATs and KDACs that, respectively, add and remove the acetyl group from the ε-amino side chain of lysine (K) [10, 51, 52]. Acetylation can influence protein functions by neutralizing the positive charge of lysine [10, 51–53]. Intriguingly, several studies have reported that lysine acetylation can also take place in a non-enzymatic manner in the mitochondria where has a high concentration of acetyl-CoA and an elevated pH that leads to the deprotonation of lysine [54–56]. Hundreds of acetylation sites have been identified in human breast cancer MDA-MB-231 cells by using proteomic techniques [57]. The effects of protein acetylation in promoting or inhibiting breast cancer may be substrate- and modification site-specific [58–60].

#### Protein acetylation plays an oncogenic role in breast cancer

Several acetylated proteins lead to poor prognosis by promoting the progression of breast cancer (Additional file 1: Table S1). KATs and KDACs regulate various pathophysiological processes and play oncogenic roles via different functional mechanisms in breast cancer. The potential molecular mechanisms of the aberrant acetylated proteins that promote the oncogenesis and progression of breast cancer can be divided into three parts.

**Fig. 2:**
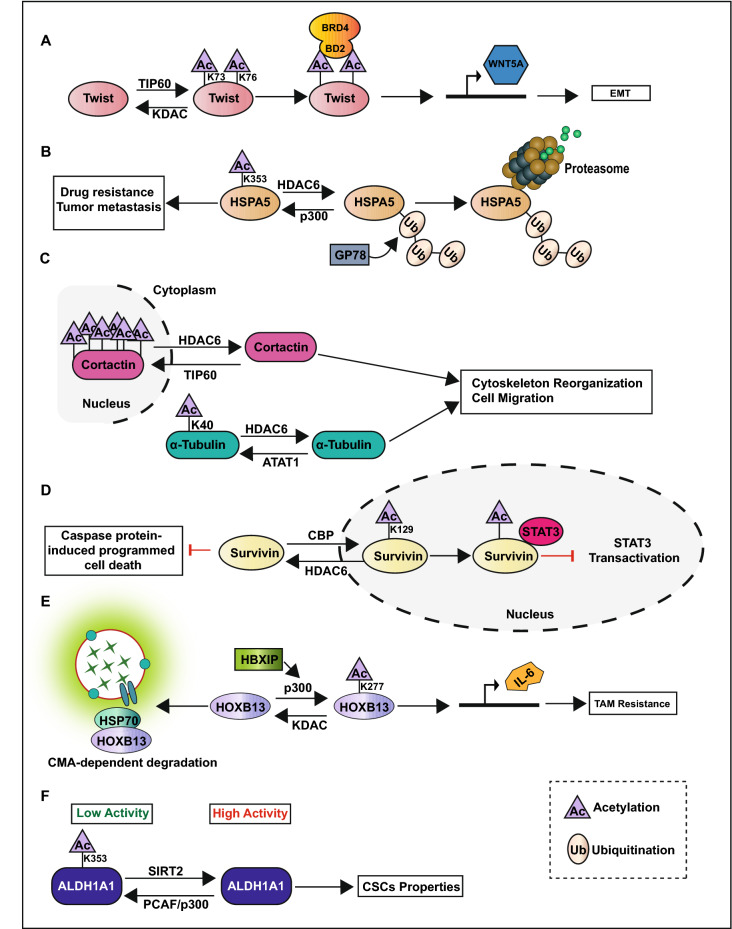
Pathophysiological processes regulated by protein acetylation in breast cancer

Firstly, acetylation promotes breast cancer metastasis. For instance, Twist, a well-known transcription factor involved in EMT, can be aberrantly activated through acetylation at K73 and K76 by 60 kDa Tat-interactive protein (TIP60) to induce its interaction with the second bromodomain (BD2) of bromodomain-containing protein 4 (BRD4) [28]. The interaction of Twist and BRD4 at the enhancer and promoter of WNT5A promote the expression of WNT5A, which leads to the activation of WNT signal pathway to accelerate EMT and tumorsphere formation in basal-like breast cancer cells (Fig. 2A) [28]. Acetylation of RelA/p65, a subunit of nuclear factor-kappa B (NF-κB), by p300 at K218, K221, and K310 activates NF-κB signal pathway and promotes the transcription of interleukin-8 (IL-8) to facilitate angiogenesis and tumor metastasis [61]. Heat-shock protein 5 (HSPA5) is regarded as a marker of poor prognosis in breast cancer due to its role in promoting drug resistance and metastasis [62]. P300 also catalyzes the acetylation of HSPA5 at K353 to inhibit its degradation mediated by E3 ubiquitin-protein ligase GP78 and promotes breast cancer metastasis (Fig. 2B) [62]. Histone deacetylase 6 (HDAC6) is reported to deacetylase cell microtubule structures, such as α-Tubulin and cortactin, and increase the formation of invadopodia that promotes breast cancer cell migration and invasion (Fig. 2C) [58].

Secondly, acetylation promotes the proliferation of breast cancer cells. For example, the acetylation of the oncogene nuclear receptor coactivator amplified in breast cancer 1 (AIB1) at K276 by the males absent on the first (MOF) protein can activate the transcription factor E2F1 to promote breast cancer cell proliferation [63]. Moreover, histone deacetylase 1 (HDAC1) can interact with the DNA binding domain (DBD) and transcription activation function domain 2 (AF-2) domains of estrogen receptor α (ERα) to inhibit its transcriptional activity and increase cell proliferation and colony formation in ER positive breast cancer cells [64]. HDAC6 deacetylases survivin to promote its cytoplasmic localization and enhance tumor cell growth and survival by inactivating caspase protein-induced programmed cell death in the cytoplasm (Fig. 2D) [59].

Thirdly, acetylation inhibits the sensitivity of tumor cells to anti-tumor therapy. Oncoprotein mammalian hepatitis B X-interacting protein (HBXIP) inhibits chaperone-mediated autophagy (CMA)-dependent degradation of homeobox B13 (HOXB13) via enhancing acetylation of HOXB13 at K277 by p300, and thus induces tamoxifen (TAM)- resistance via downregulating ERα and upregulating interleukin-6 (IL-6) expressions (Fig. 2E) [65]. Sirtuin 2 (SIRT2) is induced via the NOTCH signaling pathway to deacetylate aldehyde dehydrogenase A1 (ALDH1A1) at K353 leading to the increase of its enzyme activity and the promotion of breast cancer stem cells (CSCs) properties (Fig. 2F) [66]. Microrchidia family CW-type zinc finger 2 (MORC2) is an oncogenic chromatin-remodeling enzyme that participate in DNA repair. *N*-acetyltransferase 10 (NAT10) catalyzes the acetylation of MORC2 at K767 and thus promotes DNA damage-induced G2 checkpoint arrest and decreases the sensitivity of cancer cell to DNA-damaging chemotherapy and radiotherapy [50].

#### Protein acetylation plays a role in tumor suppression in breast cancer

Several proteins regulated by acetylation show tumor-suppressing effects in breast cancer. For any particular protein, KATs and KDACs act as Yin and Yang by exerting opposing reversible actions on the regulation of acetylation (Fig. 2). For example, as mentioned previously, the subcellular localization of survivin is crucial for its function, and HDAC6 exerts oncogenic effects by deacetylating survivin to promote its cytoplasmic localization [59]. On the contrary, survivin can be acetylated by CREB binding protein (CBP) at K129 to promote its nuclear localization and act as a tumor suppressor by inhibiting the transactivation of signal transducer and activator of transcription 3 (STAT3) (Fig. 2D) [67]. Oncoprotein HSPA5 acetylated at K353 by p300 is abrogated by HDAC6, which catalyzes the deacetylation of HSPA5 to accelerate its polyubiquitination at K447 by E3 ubiquitin ligase GP78, and induce ubiquitination-mediated protein degradation (Fig. 2B) [60]. Acetylation of ALDH1A1 at K353 by p300/CBP-associated factor (PCAF) inhibits the CSCs population as well as self-renewal property of breast cancer (Fig. 2F) [66]. The acetylation of microtubule structures, such as α-Tubulin and cortactin, by α-Tubulin *N*-acetyltransferase 1 (ATAT1) and TIP60 decreases the formation of invadopodia and inhibits breast cancer cell migration and invasion (Fig. 2C) [68]. In addition, SIRT3 inhibits CSCs reprogramming by deacetylating superoxide dismutase 2 (SOD2) at K68 [69]. P300 catalyzes the acetylation of Forkhead Box O3 (FOXO3) to promote its nuclear translocation and activation, thus increasing the cytotoxicity of Lapatinib in HER2 positive breast cancer cells [70].

### Targeting protein acetylation for breast cancer treatment

The inhibitors targeting protein acetylation can be classified into three categories, namely, KAT, KDAC, and bromodomain protein (acetyl-lysine readers) inhibitors (Additional file 1: Table S2). KAT inhibitors suppress the activity of acetyltransferases and the acetylation level of proteins. However, there has been limited research on the effects of these inhibitors in breast cancer. For example, the NAT10 inhibitor, remodelin, represses the acetylation of MORC2 and increases the sensitivity of breast cancer cells to DNA-damaging chemotherapy and radiotherapy [50]. Moreover, TH1834, a selective TIP60 inhibitor, induces apoptosis and increases unrepaired DNA damage after DNA-damaging therapy in breast cancer [22, 71]. ICG-001 specifically binds to CREB and blocks the β-catenin/CBP interaction, thereby inhibiting the EMT and invasion of MCF-7 cells [72].

The second category of inhibitors, namely, KDAC inhibitors, inhibits the activity of deacetylases and increases the acetylation level of proteins. For instance, treatment with pan-HDAC inhibitor, vorinostat, may induce cell cycle arrest and cell apoptosis in breast cancer cells [73]. Tubacin is a selective HDAC6 inhibitor that prevents estradiol-stimulated cell migration in MCF-7 cells [74]. Ricolinostat is also a selective HDAC6 inhibitor that inhibits breast cancer migration and invasion [68]. However, there has been limited research on the effects of the bromodomain protein inhibitors in breast cancer. For instance, JQ1, a bromodomain and extra terminal domain (BET) inhibitor, can suppress tumorigenesis in basal-like breast cancer via inhibiting the interaction between acetylated twist and BRD4 [28].

It has been demonstrated that NOTCH, WNT, and NF-κB signaling pathways, which are widely involved in cancer cell proliferation, metastasis, stemness and anti-tumor responses, are involved in the regulation of protein acetylation on the progression of breast cancer [13, 61, 66]. In addition, acetylation can regulate ERα expression and downstream target gene transcription, thus playing a vital role in ER positive breast cancer [64, 65]. Therefore, given the crucial role of protein acetylation in the tumorigenesis and progression of breast cancer, inhibitors that target acetylation may have potential therapeutic applications. Although the study of KDAC, KAT and bromodomain inhibitors often focus on histone acetylation, recent studies [10, 75–78] also suggest that these drugs may regulate non-histone protein acetylation in breast cancer. However, further studies are required to understand the effects of these drugs.

## Protein glycosylation

### Potential mechanisms of protein glycosylation involved in promoting breast cancer progression

Glycosylation is defined as the enzymatic process that modifies proteins or lipids by sequential addition or removal of carbohydrates [49]. Different kinds of protein glycosylation, using nucleotide sugars such as uridine diphosphate *N*-acetylglucosamine (UDP-GlcNAc) and uridine diphosphate *N*-acetylgalactosamine (UDP-GalNAc) as the sugar donor have been reported in breast cancer [49, 79–85]. Among them, *O*-GlcNAcylation, mucin type *O*-glycosylation, and N-linked glycosylation are the most widely studied protein glycosylation, the cell organelles where these modifications occur and the enzymes that regulate them are detailed in Additional file 1: Fig. S1 [49, 79, 82, 83, 85]. Recent studies have shown that the upregulation of glycosylation plays an oncogenic role in breast cancer [49, 86, 87]. Thus, here, we illustrate the causes of aberrant glycosylation and the potential mechanisms underlying protein glycosylation in breast cancer (Additional file 1: Table S1) in this section.

The increase in glycosylation in breast cancer is primarily caused by three reasons. While on the one hand, high levels of protein glycosylation may be caused in tumor cells due to the high rate of glucose uptake with activated glycolysis resulting in high levels of lactic acid produced under the conditions of sufficient oxygen supply (Warburg effect) [88, 89]. On the other hand, some glycosyltransferases, such as *N*-acetylgalactosaminyltransferase 4 (GalNAc-T4), GalNAc-T14, GalNAc-T6, fucosyltransferases 4 (FUT4), FUT8, and *O*-GlcNAc transferase (OGT) [29, 90–92], which are overexpressed in breast cancer and correlated with its prognosis, may be used as novel biomarkers or combined with traditional biomarkers to improve the sensitivity and specificity for the diagnosis of breast cancer [83, 93, 94]. However, the applications as biomarkers need to be further evaluated. Moreover, the glycosylation of glycolytic enzymes plays a crucial role in promoting the metabolic remodeling and the production of nucleotide sugars. For example, the activity of phosphofructokinase 1 (PFK1), a key enzyme in the glycolysis pathway, is suppressed by *O*-GlcNAcylated at serine (Ser)529, resulting in the redirection of glucose metabolism through pentose phosphate pathway (PPP), thus increasing nucleotide metabolism [95].

Here, we discuss the potential mechanisms of protein glycosylation that promote the oncogenesis and progression of breast cancer in the following three parts.

**Fig. 3:**
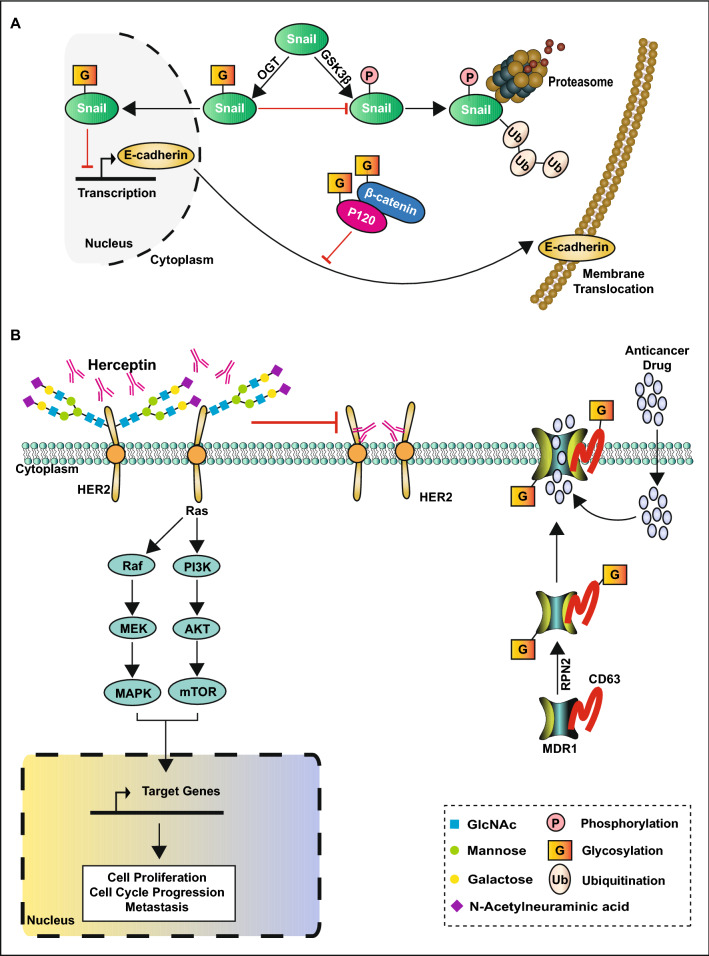
Protein glycosylation promotes the progression of breast cancer

First, glycosylation promotes the proliferation and metastasis of breast cancer cells. Glycosylation can promote cancer cell mobility and invasion by regulating the actin cytoskeleton. Cofilin, an actin-binding protein, responsible for modulating the actin dynamics to promote cell motility, is O-GlcNAcylated at Ser 108, the O-GlcNAcylation is crucial for the proper localization of cofilin in invadopodia to promote breast cancer cells mobility and invasion [96]. Furthermore, glycosylation can promote cancer cell metastasis via regulating EMT and cell adhesion. O-GlcNAcylation of snail1 at Ser112 inhibits its phosphorylation by glycogen synthase kinase-3β (GSK3β) and ubiquitin-dependent degradation, and the O-GlcNAcylated snail1 suppresses the transcription of E-cadherin [97]. The binding of p120 and β-catenin to E-cadherin is crucial for the membrane translocation and stability of E-cadherin, hyper-GlcNAcylation of p120 and β-catenin result in decreased membrane translocation of E-cadherin, thus inducing cancer cell metastasis (Fig. 3A) [82]. FUT8 plays a role in the fucosylation of transforming growth factor-β (TGF-β) serine/threonine kinase receptor I (TβRI) and II (TβRII) on the cell surface and activates the TGF-β signaling pathway to promote EMT and breast cancer cell invasion [98]. FUT8, whose expression level is positively related to a higher tumor stage and lymph node metastasis, is overexpressed in breast cancer [94]. Golgi mannosidase α-class 1A member 1 (MAN1A1) catalyzes the removal of mannose from high-mannose glycans (Additional file 1: Fig.S1). This process is crucial for the structure transformation of *N*-glycans from high-mannose structure to complex oligosaccharide chain. MAN1A1 silencing or use of the mannosidase inhibitor kifunensine [99] to reduce MAN1A1 expression significantly increases the adhesion of breast cancer cells to endothelial cells. A previous study on the clinical samples of breast cancer patients showed that the patients who had low levels of MAN1A1 were more likely to have higher tumor metastasis and shorter disease-free survival [100]. Forkhead box protein A1 (FOXA1), a transcription cofactor of ERα that promotes ERα recruitment, may be glycosylated by GalNAc-T4 to enhance protein stability and regulate the estrogen network. Knockdown of GalNAc-T4 decreases the expression of cyclin D1 and induces cell cycle arrest [101]. Furthermore, the O-glycosylation of ERα at Ser573 by GalNAc-T6 is crucial for the nuclear localization and downstream target transcription of ERα to promote cell proliferation [102]. Mucin 1 (MUC1) promotes EMT by regulating various EMT-related signaling pathways, such as the NF-κB, TGF-β, and STAT3 pathways [103]. Moreover, the overexpression of GalNAc-T6 can glycosylate and sustain the stability of MUC1, leading to proliferation and cell adhesion reduction of cancer cells [104].

Next, glycosylation inhibits the sensitivity of tumor cells to anti-tumor therapy. Treatment with PUGNAc (OGA inhibitors) and glucosamine to increase the level of O-GlcNAcylation can significantly reduce the expression of ERα in ER positive breast cancer cells and protect the cells from tamoxifen-induced death [105]. On the contrary, the inhibition of OGT by siRNA potentiates the expression of p21 and early growth response gene 1 (Egr1) induced by tamoxifen to promote cell cycle arrest and cell death [105]. These data suggest that the inhibition of O-GlcNAcylation might help to improve the efficacy of anti-estrogen therapy in breast cancer. However, the underlying mechanisms are not yet clearly understood [105]. N-linked glycosylation regulates the responsiveness of breast cancer cells to chemotherapy. The *N*-glycosylation of HER2 protein at asparagine (Asn) 68/124/187/259/530/571/549 can inhibit its binding with Herceptin and activate mitogen-activated protein kinase (MAPK) and PI3K-Akt signaling pathways to promote breast cancer progression (Fig. 3B). A reduction in cell surface glycosylation along with increased sensitivity to Herceptin and doxorubicin has been reported following tunicamycin treatment [106]. Ribophorin II (RPN2), a part of the *N*-oligosaccharyltransferase complex, promotes breast cancer malignancy by regulating the glycosylation of tetraspanin protein CD63 and multidrug resistance protein 1 (MDR1). The glycosylated CD63 and MDR1 can interact with each other and co-localized in the cell membrane and play an essential role in reducing the concentration of anticancer drugs in cancer cells by facilitating the efflux of the anticancer drug out of cells, thus leading to drug resistance and cancer invasiveness (Fig. 3B) [107]. Collectively, these studies highlight the potential of glycosylation inhibitors in combination with other anticancer therapies in the treatment of breast cancer.

**Fig. 4:**
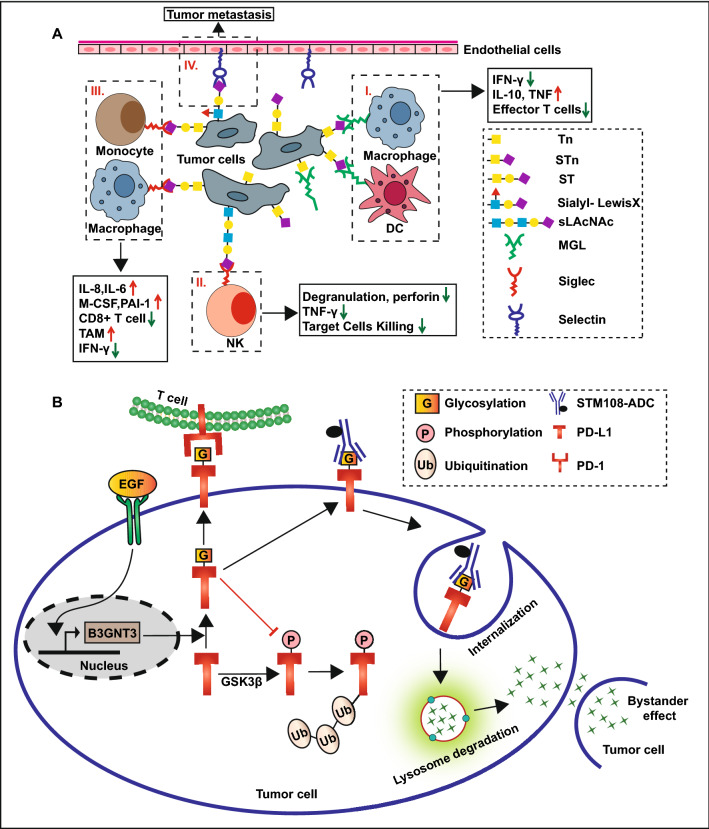
Protein glycosylation regulates the immune microenvironment and anti-tumor immune response

Lastly, glycosylation promotes tumor growth by regulating the immune microenvironment and antitumor immune response. Abnormal glycosylation of tumor-related epitopes (such as hypersialylation in breast cancer) result in the altered interaction with lectins expressed in immune cells, which activate the inhibitory signals in the immune cells and lead to tumor immune suppression [85, 108, 109]. In breast cancer, the upregulation of Tn and sialylated Tn (STn) glycans can be recognized and bound by macrophage galactose-specific lectin (MGL) expressed in the macrophages and dendritic cells [110]. This glycosylation-dependent interaction drives an immune inhibitory program that decreases the production of IFN-γ and increases the expression of IL-10 and TNF, thus decreasing the effector T cell proliferation and increasing effector T cell apoptosis (Fig. 4A, I) [110]. The presence of cell surface sialic acids have been referred to as ‘self-associated molecular patterns (SAMPs)’ that are recognized by sialic acid-binding immunoglobulin-like lectins (siglecs) expressed by immune cells to produce signals that negatively regulate the immune system [111]. Similar to the immune checkpoint receptor programmed cell death 1 (PD-1), most siglecs contain a cytosolic immunoreceptor tyrosine-based inhibitory motif (ITIM) that can recruits SH2-containing inositol phosphatase (SHP) to halt the kinase phosphorylation cascade in immune cell, and thus inhibit the activity of immune cell [112, 113]. For example, the binding of sialylated *N*-acetyl-d-lactosamine (LacNAc) to siglec-7 on the NK cells can protect the cancer cells from NK cell cytotoxicity by decreasing the production of IFN-γ and other cytotoxic molecules (Fig. 4A, II) [114]. The binding of mucin 1-sialylated core 1 (MUC1-ST) to siglec-9 on monocytes and macrophages can induce the release of factors such as IL-8, IL-6, macrophage colony-stimulating factor (M-CSF), and plasminogen activator inhibitor-1 (PAI-1), that promote tumor growth and induce macrophages to develop a tumor-associated macrophages (TAM) phenotype (Fig. 4A, III) [115, 116]. In addition, the interactions between sialyl-Lewis X glycans on the leukocytes and selectins on the endothelial cells are crucial for immune cell trafficking [117]. Similarly, the cancer cells that overexpress sialyl-Lewis X glycans can bind to the endothelial cells and promote tumor metastasis by this mechanism (Fig. 4A, IV) [117]. Thus, targeting this aberrant glycosylation may be a potential novel therapy for breast cancer treatment [118–120]. For example, the sialidase conjugate trastuzumab can desialylate tumor cells and inhibit the interaction between the sialylated glycans and inhibitory siglec receptor on the NK cell to enhance NK cell cytotoxicity [119, 120]. Tumor microenvironment induces programmed cell death-ligand 1 (PD-L1) expressed in tumor cells. The binding of PD-L1 to PD-1 expressed in the immune cell can protect the tumor cells from the immune cell attack [121]. *N*-glycosylation of PD-L1 helps maintain its protein stability by antagonizing the binding of GSK3β and phosphorylation-induced proteasome degradation [122]. Previous studies have reported that β-1,3-*N*-acetylglucosaminyltransferase (B3GNT3), an enzyme which is transcriptionally activated by EGF, catalyzes the *N*-glycosylation of PD-L1 at Asn192 and Asn200 in TNBC, thus promoting its interaction with PD-1 and inducing immune suppression and T cell exhaustion [49, 123]. The STM108 antibody can specifically recognize the Asn192 and Asn200 glycosylation sites of PD-L1 and induce PD-L1 internalization and degradation to reactivate T cells. Moreover, the antibody–drug conjugate, STM108-ADC, induces potent drug-induced cytotoxic activities and bystander effects to kill TNBC cells both in vivo and in vitro (Fig. 4B) [123]. This implies that targeting protein glycosylation might be a potential way to enhancing the effects of immune checkpoint therapy in TNBC and need further verification in clinical trials.

### Targeting protein glycosylation for breast cancer treatment

Currently, these drugs and inhibitors target at protein glycosylation (Additional file 1: Table S2) for breast cancer treatment can be divided into the following two categories. The first category is monosaccharide analogs. As several cell membrane and secretory proteins are glycoproteins, the monosaccharide analogs may participate in the glycosylation pathway to alter the glycan structure and disrupt the elongation of the oligosaccharide chains [86, 124]. Consequently, they may affect the oncogenic functions of the corresponding glycoproteins. The monosaccharide analogs include the glucose analog 2-Deoxy-D-glucose (2-DG) [86], GalNAc analog 2-KetoGal [124], and fucose analog 6-Azidofucose [125]. The second category involves glycosyltransferase and glycoprotein inhibitors that targets at high levels of glycosylation in breast cancer. For example, the reduction in cell surface glycosylation together with the increased sensitivity to Herceptin and doxorubicin has been found following tunicamycin treatment [106]. Ginsenoside Rg3 inhibits the expression of FUT4 and inhibits fucosylation modification [126]. Epigallocatechin gallate (EGCG) can inhibit the production of MUC1 and thereby suppress breast cancer metastasis [80, 127].

In consideration that dysregulation of diverse glycosyltransferase results in upregulation of glycoprotein during breast cancer oncogenesis and progression, many glycoproteins may be viewed as biomarkers for breast cancer diagnosis. To date three serum glycoproteins including cancer antigen (CA 15–3) and CA 27–29 that encoded by MUC1 gene, and carcinoembryonic antigen (CEA) are regarded as traditional biomarkers used for clinically detection and monitor breast cancer occurrence and recurrence through serum glycoprotein immunoassays [49, 81, 128]. However, it is significant to find new markers that specific for breast cancer due to the lake of sensitivity and specificity of traditional glycoprotein biomarkers. Recently, many researchers are increasingly interest to glycosylated biomarkers within exosomes or extracellular vesicles that derived from cells and biofluids. This novel field termed liquid biopsies [129, 130]. Liquid biopsies may have the potential to be used in breast cancer as these vesicles also contain aberrant glycoproteins.

## Protein sumoylation

### Potential mechanisms of protein sumoylation involved in breast cancer

Sumoylation is a three-step enzymatic cascade reaction analogous to ubiquitination catalyzed by SUMO-E1 activating enzyme, SUMO-E2 conjugating enzyme, and SUMO-E3 protein ligases to covalently attach the small ubiquitin-related modifier (SUMO) proteins to the lysine residues of the target proteins [9, 131]. Sumoylation is a reversible modification that can be deSUMOylated by SUMO-specific proteases (SENPs) [9, 17, 25]. The dysregulation of protein sumoylation may either promote or suppress the progression of breast cancer [17, 23, 132–135]. Hence, here, we discuss the molecular mechanisms underlying sumoylation in breast cancer.

#### Protein sumoylation plays an oncogenic role in breast cancer

In general, protein sumoylation may promote breast cancer tumorigenesis and progression by accelerating cell cycle transition and proliferation, facilitating tumor cell EMT and migration. These mechanisms are described in detail in this section.

**Fig. 5:**
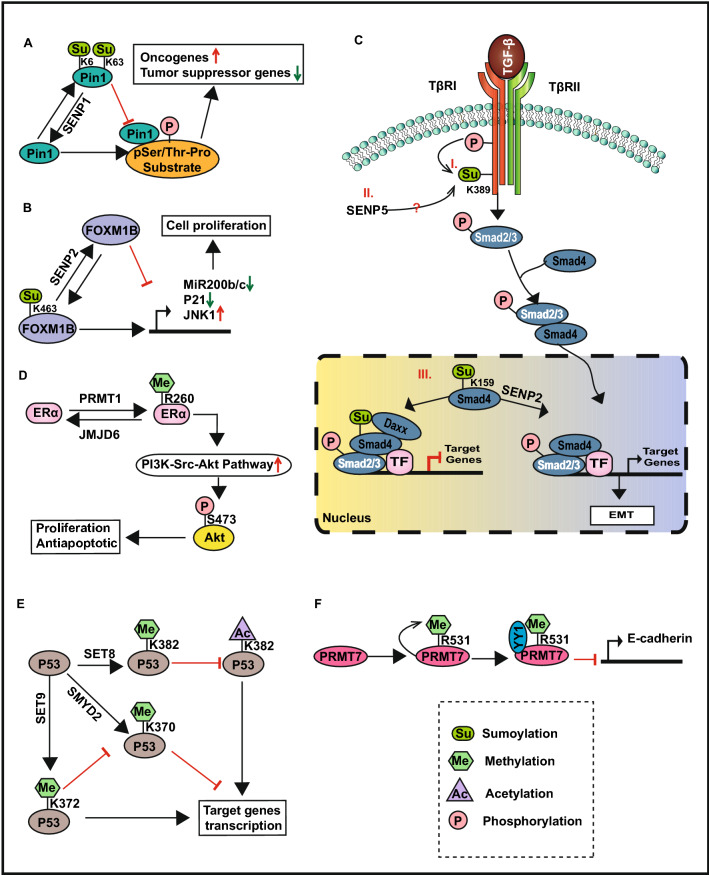
The potential mechanisms of sumoylation and methylation regulating breast cancer

Firstly, protein sumoylation accelerates cell cycle transition and proliferation of breast cancer cells. For example, BRCA1, a well-known breast cancer susceptibility gene associated with DNA damage repair, cell cycle regulation, and sustained chromosomal genomic stability, may be sumoylated at K32 and K1690 in the ER positive breast cancer cells to induce G0/G1 phase transition and oxidative stress response [136]. Further, The sumoylation of the transcriptional co-repressor KRAB domain-associated protein 1 (KAP1) at K554, K779, and K804 attenuates the acetylation and augments the methylation of H3K9 at the p21 promoter, thus repressing the expression of p21 and promoting MCF-7 cell proliferation [137]. The upregulation of sumoylation-related enzymes, such as UBC9 and PIAS1, has been shown in several breast cancer tissue arrays [138–141]. UBC9, the sole SUMO E2 conjugating enzyme, may induce tumor cell resistance to chemotherapy via upregulating expression of anti-apoptotic protein Bcl-2 and is correlated with poor clinical prognosis in breast cancer [139, 140, 142]. DeSUMOylation of protein interacting with never in mitosis A (NIMA)-1 (Pin1) at K6 and K63 by SENP1 promotes its protein activity and interaction with the substrate, thereby inducing malignant cell transformation (Fig. 5A) [143]. MiR200 b/c acts as a tumor suppressor by upregulating the expression of E-cadherin [144, 145]. Sumoylation of the transcription factor Forkhead Box Protein M1 (FOXM1B) at K463 inhibits the expression of miR200 b/c and p21, thus activating the expression of JNK1 and promoting the proliferation of MCF-7 cells (Fig. 5B) [146].

Moreover, protein sumoylation facilitates breast cancer cell EMT and migration. The sumoylation of talin, a key component of focal adhesions (FAs) that accelerating cancer cell migration by linking the cytoskeleton to the extracellular matrix, at K2445 and K841 positively regulates FAs disassembly and promotes MDA-MB-231 cell migration [147]. The sumoylation of TβRI at K389 in response to TGF-β depends on its kinase activity and phosphorylation modification. Further, the sumoylation of TβRI enhances its interaction with Smad2/3 and promotes the phosphorylation of Smad3, consequently activating TGF-β-Smad signaling pathway to promote cancer cell metastasis (Fig. 5C I) [148]. Interestingly, a previous study also demonstrated that SENP5 promotes breast cancer invasion by sustaining the sumoylation of TβRI (Fig. 5C II), whose expression is negatively correlated with the prognosis of breast cancer patients [134]. However, whether this contradiction is attributed to the interaction between phosphorylation and sumoylation or to the difference in the modified site is unclear and needs to be further investigated [134]. SENP2 facilitates TGF-β-Smad4 signaling pathway by desumoylating Smad4 at lys159 to promote EMT and cell migration in TNBC cells and sustain cancer stem cell properties (Fig. 5C III) [149]. The high expression of SENP2 consequently leads to poor prognosis in TNBC patients [149].

#### Protein sumoylation plays a role in tumor suppression in breast cancer

For any specific protein, similar to acetylation, reversible protein sumoylation can play opposing roles as an oncogene and a tumor suppressor. For example, the sumoylation of Pin1 at lys6 and lys63 suppressed its ability and oncogenic function (Fig. 5A) [143]. Sumoylation of Smad4 at K159 promotes its interaction with the transcriptional corepressor Daxx to repress the transcriptional activity of Smad4, thereby inhibiting the TGF-β-Smad4 signaling pathway and playing the role of a tumor suppressor in breast cancer (Fig. 5C III) [150]. SENP2 deSUMOylates FOXM1B at K463 and thus upregulates the expression of miR200 b/c and p21 to reduce the proliferation and migration of MCF-7 breast cancer cells (Fig. 5B) [146]. In addition, SENP2 acts as an ERα transcriptional corepressor by recruiting HDAC3 to the promoter of ERα, it also influences the cell cycle G1/S transition and inhibits the proliferation of ER-positive breast cancer MCF-7 cells [151].

### Targeting protein sumoylation for breast cancer treatment

However, since this modification has been identified only recently, the inhibitors targeting sumoylation in breast cancer are somewhat limited (Additional file 1: Table S2). Recent studies have identified two pharmacological inhibitors of the SUMO pathway, namely, ginkgolic acids C15:1 (GA C15:1) that interact with SUMO E1 activating enzymes to abrogate the formation of the E1-SUMO1 complex [152], and 2-D08, which suppresses sumoylation by inhibiting the transfer of SUMO from SUMO E2 conjugating enzyme to target substrate [153]. These two inhibitors play a pivotal anti-cancer role in breast cancer cell lines, such as MDA-MB-231, MCF-7, and BT474, not only by inducing the expression of autophagy modulator Tribbles pseudokinase 3 (TRIB3) and the transcription of various autophagy-related genes, such as ATG1, ATG7, and BECN1 to accelerate autophagy-dependent cancer cell death, but also by inhibiting the sumoylation of RAC1 (a member of Rho GTPase family), and thus suppress the activation RAC1 and repress the RAC1-mediated cell migration and invasion [154, 155]. Moreover, Triptolide, a component extracted from the Chinese herb, *Tripterygium wilfordii Hook F*, acts as a natural SENP1 inhibitor that downregulates the expression of the androgen receptor (AR) and c-Jun to restore the balance between sumoylation and deSUMOylation and consequently inhibits prostate cancer [156]. Considering that SENP1 also overexpressed in breast cancer [143], Triptolide may potentially be used for the treatment of breast cancer. However, its specific anti-cancer function has yet not been verified in breast cancer.

The SUMO gene was first discovered in 1995 [157], and a recent proteomics study indicated that at least 1000 human proteins were modified by SUMO proteins [16]. Considering that this modification is somewhat newly discovered, our understanding of protein sumoylation is somewhat limited. The mechanism by which sumoylation is involved in the progression of breast cancer remains to be further studied, for example, whether sumoylation is participated in the anti-tumor immune response of breast cancer is still unknown. In addition, inhibitors targeting sumoylation remains to be explored.

## Protein methylation

### Potential mechanisms of protein methylation involved in breast cancer

The process in which protein methyltransferases transfer the methyl group from s-adenosyl methionine (SAM) to the side chains of amino acid residues, such as arginine, lysine, glutamate, and cysteine, consequently producing a methylated residue and s-adenosyl homocysteine (SAH), is called as protein methylation [8, 15, 158–164]. The methylation of lysine and arginine residues on proteins, catalyzed by PKMT and PRMT, respectively, is the prominent and universal types of methylation modification occurring in breast cancer [8, 15]. Lysine methylation is a reversible modification, and several protein lysine demethylases (PKDMs), as their name suggests, possess the lysine demethylase activity [15, 165, 166]. In the case of arginine demethylation, the data on the sole putative arginine demethylase JMJD6 is controversial [167, 168]. Therefore, a bona fide arginine demethylase is yet to be identified. In this section, we present the underlying mechanisms of protein methylation in breast cancer carcinogenesis and metastasis [19, 48, 165, 166, 169].

#### Protein methylation plays an oncogenic role in breast cancer

Several studies have confirmed that PRMT1, PRMT2, PRMT3, and PRMT7 are overexpressed in breast cancer [159, 170–174]. Moreover, co-activator associated arginine methyltransferase 1 (CARM1, also known as PRMT4) is overexpressed in metastatic breast cancer as opposed to normal breast tissues [173, 175]. Among the numerous PKMT and PKDM that have been identified so far, SET6, SMYD2, SMYD3, LSD1, and KDM2A have been shown to regulate breast cancer [48, 163, 165, 166]. The mechanisms protein methylation participates in breast cancer tumorigenesis and progression are described in further detail in this section.

Methylation activates oncogenic signaling pathways to accelerate breast cancer progression. For example, the methylation of BRG1-associated factor 155 (BAF155), a core subunit of chromatin remodeling complex SWI/SNF, at R1064 by CARM1 regulates the expression of target genes in the c-MYC pathway, and thus accelerates the progression of breast cancer, as previously shown in both, in vivo and in vitro* assays* [173]. PRMT1 catalyzes ERα methylation at R260 within the DNA binding domain during rapid estrogen signaling, leading to the activation of the downstream PI3K-Src-Akt signaling pathway, thus promoting the phosphorylation of Akt at Ser473 and resulting in cancer cell proliferation and antiapoptotic effects (Fig. 5D) [171]. The dimethylation of Akt at R391 by PRMT5 is essential for its kinase activity and breast cancer tumorigenesis [176]. Moreover, LSD1 catalyzes the demethylation of ERα at K266 to promote ERα signaling and cell proliferation [177]. KDM2A activates the Notch signaling pathway to enhance the stemness of breast cancer cells [165].

Furthermore, protein methylation regulates the anti-tumor effects of tumor suppressor proteins. For instance, programmed cell death protein 4 (PDCD4) is methylated at R110 by PRMT5 to inhibit its anti-tumor properties, and the co-expression of PDCD4 and PRMT5 generates a tumor-promoting phenotype in an orthotopic breast cancer model [172]. Several previous studies have demonstrated that the activity of p53, a well-known tumor suppressor protein, is regulated by protein methylation [170, 178–180]. P53 monomethylated at K370 by SMYD2 represses its antitumor ability, thus inhibiting p53-mediated apoptosis [179]. However, K372 monomethylated by SET9 inhibits the interaction between SMYD2 and p53 and thus increases p53 stability and its target gene transcription [8, 163]. In addition, the p53 monomethylated at K382 by SET8 represses its acetylation and transcriptional activity [180]. However, future research is required to analyze whether the methylation of p53 plays a role in breast cancer (Fig. 5E).

Finally, protein methylation facilitates breast cancer cells EMT and migration. WD repeat domain 5 (WDR5), a core subunit of the histone methyltransferase (HMT) complex positively correlated with a higher clinical stage and histological grade of tumor [181, 182], is methylated by SET6 at K207 and K325 in breast cancer cells to promote cell proliferation and migration [48]. KDM2A interacts with RelA to co-occupy at the promoter region of tet-eleven translocation 2 (TET2) and repress the expression of its target genes including epithelial cell adhesion molecule (EpCAM) and E-cadherin, thus accelerating EMT and angiogenesis, TNBC patients with overexpressed KDM2A often related to worse survival [166]. Intriguingly, some PRMTs undergo automethylation spontaneously, the automethylation of PRMT7 at R531 in the C-terminal of the protein accelerates the interaction with the transcription factor Yin-Yang 1 (YY1) and is vital for its recruitment to the promoter region of E-cadherin to inhibit transcription, thus promoting EMT and breast cancer cell migration and invasion (Fig. 5F) [19].

#### Protein methylation plays a role in tumor suppression in breast cancer

The reversible modifications of protein methylation, i.e. methylation and demethylation, of the specific protein at the same site may have contrary effects. For example, the demethylation of ERα at R260 by JMJD6 inhibits the activation of the PI3K-Src-Akt signaling pathway, thus inhibiting breast cancer cell proliferation (Fig. 5D) [171]. On the other hand, the methylation of ERα at K266 attenuates the chromatin recruitment of ERα and its target gene expression [177].

### Targeting protein methylation for breast cancer treatment

Pharmacological inhibitors targeting the protein methylation have a crucial role in cancer treatment (Additional file 1: Table S2). For example, GSK591 is a selective PRMT5 inhibitor that suppresses breast CSCs proliferation and self-renewal [183]. GSK3326595 is also a selective PRMT5 inhibitor that inhibits the activation of Akt to sensitive breast cancer cells to etoposide and cisplatin [176]. DC_C66 and DC_C11 are cell membrane permeable CARM1 inhibitors that effectively suppress the proliferation of MCF-7 cells by competitively occupying the binding site of the substrate [184]. PKMT inhibitor MS1943 inhibits the proliferation of multiple TNBC cells [185]. Daminozide is a KDM2A inhibitor that can inhibit cancer cell stemness and enhance the sensitivity and cytotoxicity of cisplatin in MDA-MB-231 cells [165].

A majority of the previous studies on methylation mainly focus on DNA and histone methylation [30, 32, 42, 44], non-histone methylation, as a burgeoning field, the studies and literatures are somewhat limited in breast cancer. Thus, considering several methylases and demethylases discovered so far, it may be worthwhile to focus further studies in this field to discover potential breast cancer therapies. For instance, methylation of ERα at R260 by PRMT1 promoting the phosphorylation of Akt at Ser473 and the activation of PI3K-Src-Akt signaling pathway [171]. Moreover, the dimethylation of Akt at R391 by PRMT5 is essential for its kinase activity [176]. Therefore, whether there is a synergistic regulatory relationship between PRMT1 and PRMT5, or whether there exists a positive feedback between the phosphorylation and methylation of Akt, deserves further study.

## Protein ubiquitination

### Potential mechanisms of protein ubiquitination involved in breast cancer

Protein ubiquitination is a multi-step process sequentially catalyzed by enzyme complexes consisting ubiquitin-activating enzymes (E1), ubiquitin-conjugating enzymes (E2), and ubiquitin ligases (E3) (Additional file 1: Fig. S2) [24]. Among these enzymes, the E3 ubiquitin ligases selectively interact with specific target proteins and play a corresponding role in cellular physiology [24, 186, 187]. E3 ubiquitin ligases can be divided into three categories, including RING E3s, homologous to the E6AP carboxyl terminus (HECT) E3s, and RING-in-between-RING (RBR) E3s (Additional file 1: Fig. S2) [187]. Different from classic E3s, including HECT and RING E3 ligases, which been regarded as destructive ubiquitin ligases and well-studied in breast cancer [27, 46, 188–195], atypical ubiquitin ligase of the RBR E3 ligases is prone to catalyze mono-ubiquitination or linear poly-ubiquitination of the substrates. However, it does not lead to the degradation of the substrate but plays a vital role in signal transduction and regulation of gene transcription [7, 187]. In addition, over 600 E3 ligases have been identified in humans so far, whereas only about 12 RBR E3 ligases have been reported [187]. Due to the special functions and the limited number of RBR E3 ligases, the pathways and regulatory mechanisms underlying the role of RBR E3 ligases in breast cancer carcinogenesis and evolution are illustrated in detail in this section.

#### RBR E3 ligases play an oncogenic role in breast cancer

Ring finger protein 31 (RNF31, also named HOIP), Ran Bp-type and C3HC4-type zinc finger-containing protein 1 (RBCK1), and Ariadne homolog 1 (ARIH1) are members of RBR E3 ligases that are up-regulated in breast cancer [18, 196, 197].

**Fig. 6:**
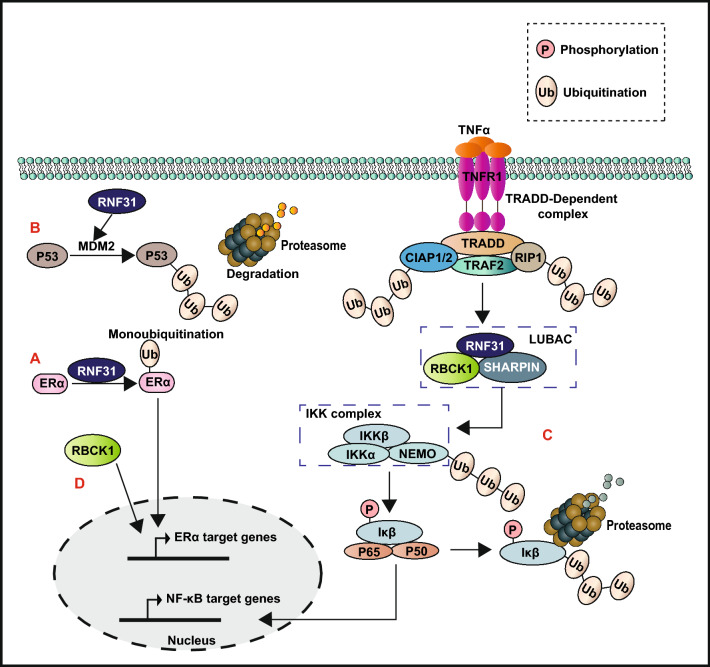
The proposed model for the effects of RNF31 and RBCK1 in breast cancer

RNF31 was originally cloned from breast cancer cells and was identified to be highly expressed in breast cancer tissues compared to the adjacent normal tissues [196]. Recent studies have shown that RNF31 may function as a breast cancer oncogene by any of the following mechanisms. RNF31 acts as an oncogene by catalyzing the monoubiquitination of ERα to increase its stability and promotes the transcription of the ERα signal downstream oncogenic proteins (cyclin D1 and c-myc) to accelerate cell cycle transition and cancer cell proliferation (Fig. 6A) [196]. Moreover, RNF31 inhibits the ubiquitination of MDM2 through an unknown mechanism to accelerate p53 degradation, which resulting in chemotherapy resistance by inhibiting p53 induced cell apoptosis (Fig. 6B) [196, 198]. In addition, RNF31 and RBCK1 are the vital components of linear ubiquitin assembly complex (LUBAC), that mediate the linear polyubiquitination of the inhibitor of κB Kinase (IKK) γ (NEMO), thereby activating the IKK complex and facilitating NF-κB signaling (Fig. 6C) [196]. RBCK1 also increases the transcription of ERα and cyclin B1 by recruitment to the ERα promoter to enhance cancer cell proliferation (Fig. 6D) [199]. ARIH1 may facilitate the removal of damaged mitochondria to protect breast cancer cells from chemotherapy-induced death [197].

#### RBR E3 ligases play a role in tumor suppression in breast cancer

RNF144A and Parkinson protein 2 (PARK2, also known as Parkin) are members of the RBR E3 ligase family that have been identified as tumor suppressor genes in breast cancer, their low expression levels in breast cancer may be attributed to hypermethylation in their promoter [200, 201]. RNF144A ubiquitinates and degrades the DNA-dependent protein kinase catalytic subunit (DNA-PKcs) and poly (ADP-ribose) polymerase 1 (PARP1) to inhibit the repair of DNA damage via non-homologous end joining (NHEJ) and base excision repair (BER) pathways, respectively [202, 203]. Thus, it promotes cell death and acts as a tumor suppressor. PARK2-mediated HIF-1α ubiquitination at K477 and degradation inhibits breast cancer metastasis [204]. PARK2 also decreases the expression of Cyclin-dependent kinase 6 (CDK6) and negatively regulates the proliferation of breast cancer cells [205]. In addition, Parkin binds to microtubules and increases the interaction between paclitaxel and the microtubule, thus enhancing paclitaxel sensitivity in breast cancer [197, 206].

### Targeting protein ubiquitination for breast cancer treatment

Some small molecule inhibitors targeting ubiquitination may be potential novel therapies for breast cancer (Additional file 1: Table S2). For example, WP1130 has been shown to effectively promote chemotherapy-induced tumor cell death by inhibiting USP9X [46]. ML364, a USP2 inhibitor, induces cyclin D1 degradation and causes cell cycle arrest in MCF-7 cells [207]. Nutlin-3 inhibits MDM2-dependent P53 ubiquitin degradation and causes cell cycle G1 arrest [208]. Similarly, SP-141 promotes MDM2 auto-ubiquitination and degradation to suppress breast cancer [209]. Furthermore, traditional Chinese medicines have attracted increasing attention in recent years. Celastrol, a component extracted from the Chinese herb *Tripterygium wilfordii Hook F*, is a proteasome inhibitor that represses the degradation of tumor suppressor proteins to promote cancer cell apoptosis [210]. This anti-cancer activity has been demonstrated in prostate cancer and may also have an influence on breast cancer oncogenesis, however, further studies are required to confirm its effects in breast cancer.

In general, several substrate ubiquitination sites still remain unidentified, and the function of RBR E3 ligases has been studied mainly through gene silencing [196, 198, 211]. Considering the vital role of RBR E3 ligases in the regulation of breast cancer, small-molecule inhibitors that target these enzymes may have a therapeutic potential in the treatment of breast cancer.

## Other PTMs involved in breast cancer

In addition to the PTMs mentioned above, some other rare PTMs, such as citrullination and palmitoylation, have also been reported in breast cancer [26, 212, 213]. Citrullination is defined as a process of deimination of arginine and conversion to citrulline (Cit), thus replacing the positively charged arginine by uncharged citrulline catalyzed by peptidyl arginine deiminases (PADs) [214, 215]. Peptidyl arginine deiminase 2 (PADI2) is overexpressed in breast cancer and is associated with tumorigenesis and progression [213]. PADI2 regulates RNA polymerase II (RNAP2) transcriptional activity by catalyzing the deamination of R1810 to Cit1810, thus promoting gene transcription and cell proliferation in breast cancer [215, 216]. PADI2 serves as a mediator of the EGF-PI3K signaling pathway to accelerate tumor cell invasion and migration by activating the components of the Rho family, including Rho, Rac, and Cdc42 [26]. The citrullination of GSK3β at R3 is important for its nuclear localization and the inhibition of the TGF-β signaling pathway, thus inhibiting the EMT of breast cancer cells [47]. Protein palmitoylation (also known as protein S-acylation) is a reversible PTM that is catalyzed by protein acyltransferases (PATs) and acylprotein thioesterases (APTs) to either link or remove a palmitate group to cysteine (Cys) residues [217–219]. Palmitoylation of the adhesion protein CD44 at cysteine (Cys) 286 and Cys295 increases its raft affiliation but decreases its interaction with the migratory binding partner ezrin, thus inhibiting breast cancer cell migration [220].

### Targeting protein citrullination and palmitoylation for breast cancer treatment

Both, the first-generation pan-PADs inhibitor, Cl-Amidine [214] and the more potent second-generation inhibitor, BB-Cl-Amidine [221] can maintain the integrity of the basement membrane and suppress the proliferation and migration of breast cancer cells in vivo as well as in vitro* assays* [26]. D-Cl-amidine, a selective PAD1 inhibitor, decreases cell viability in MDA-MB-231 cells [185]. Moreover, curcumin inhibits the migration of breast cancer cells by repressing the palmitoylation of integrin β4 (ITG β4) and ITG β4-dependent cell migration [222].

## The crosstalk between PTMs

Most proteins are modified by multiple PTMs, and the different kinds of protein PTMs can interact with each other. Such crosstalk between the PTMs can integrate diverse signals and vastly increase their regulatory potential.

**Fig. 7:**
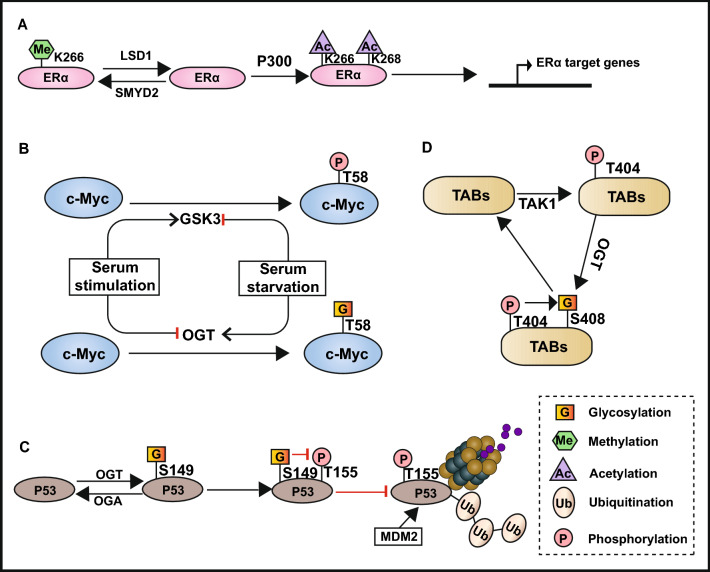
The crosstalk between PTMs

Firstly, different kinds of PTMs may take place at different amino acid such as lysine, arginine, threonine (T), serine, asparagine, tyrosine, and cysteine [7, 9, 10, 15, 38, 223]. However, lysine serves as the most universal target protein amino acid residue that can be regulated by several PTMs, such as ubiquitination [24], sumoylation [9, 131], methylation [15, 163], and acetylation [10, 224]; these modifications may influence each other and compete for the same lysine site. For example, SUMO covalently attached to the lysine site is generally used to inhibit the conjugation of ubiquitin to protect the target proteins from ubiquitin-mediated degradation [9, 131, 225]. Methylation of ERα at K266 by SMYD2 inhibits its acetylation at K266/268 catalyzed by p300, which can be reversed by demethylase LSD1 and promotes the expression of ERα target genes (Fig. 7A) [177]. HDAC6 catalyzes the deacetylation of HSPA5 at K353 and accelerates its polyubiquitination at K447, thus inducing ubiquitination-mediated protein degradation (Fig. 2B) [60].

Moreover, many protein post-translational modifications are involved in the progression of breast cancer by regulating the activity of signaling pathways [61, 66, 67, 98, 106, 149, 171], the activation of most signaling pathways such as NF-κB, TGF-β-Smad4 and PI3K-Akt is a cascade of phosphorylation modification [61, 98, 106, 149]. Therefore, there are many crosstalk between phosphorylation and other PTMs in breast cancer, for example, the crosstalk between methylation and phosphorylation; methylated ERα at R260 by PRMT1 triggers the PI3K-Akt signaling pathway to stimulate the downstream target proteins to undergo phosphorylation modification and resulting in breast cancer cell proliferation and antiapoptotic effects (Fig. 5D) [171]. Dimethylation of Akt at R391 promotes its phosphorylation at T308 and thereby promoting PI3K-Akt activation to promote breast cancer progression [176]. The crosstalk between sumoylation and phosphorylation; The Ras-ERK2 signaling pathway mediates the phosphorylation of CCAAT/enhancer binding protein beta1 (C/EBPbeta1) at Thr235 that accelerates the sumoylation of C/EBPbeta1 to facilitate breast cancer cell escape from oncogene-induced senescence [132]. Forkhead Box Protein P3 (FOXP3) is recruited in the promoter region of UBC9 to promote its transcription and translation, however, the loss of phosphorylation on tyrosine 342 or the loss of acetylation and/or ubiquitination on K263 in FOXP3 suppress its role in promoting UBC9 expression and sumoylation [135]. In addition, there is much crosstalk between O-GlcNAcylation and phosphorylation; for example, T58 of the transcription factor c-Myc can be both a target for phosphorylation and O-GlcNAcylation, while serum starvation promotes O-GlcNAcylation of c-Myc, serum stimulation shows the opposite effects (Fig. 7B) [226]. The O-GlcNAcylation of p53 at Ser149 inhibits its phosphorylation at Thr155, resulting in a reduced interaction between MDM2 and p53, and consequently inhibits the ubiquitin-dependent degradation of p53 (Fig. 7C) [227]. Similarly, the O-GlcNAcylation of snail1 inhibits its phosphorylation-mediated proteasome degradation (Fig. 3A) [228]. Except for O-GlcNAcylation, the *N*-glycosylation of PD-L1 can maintain its protein stability by antagonizing the binding of GSK3β and phosphorylation-induced proteasome degradation (Fig. 4B) [122, 123]. However, the crosstalk between phosphorylation and O-GlcNAcylation is not always mutually exclusive. For instance, the O-GlcNAcylation of TGF-β activated kinase 1 binding proteins (TABs) at Ser408 is accelerated by its phosphorylation at Thr404, and the O-GlcNAcylated TABs, in turn, activate TGF-β activated kinase 1 (TAK1) and its downstream NF-κB signaling pathway; this positive feedback facilitates the migration and invasion of TNBC (Fig. 7D) [229].

Furthermore, in addition to the crosstalk between non-histone PTMs, the crosstalk also exists between histones and non-histone PTMs [50, 137]. For example, the acetylation of MORC2 at K67 by NAT10 inhibit histone phosphorylation at H3T11 and induce the transcriptional repression of CDK1 and cyclinB1 to decreases the sensitivity of cancer cell to DNA-damaging chemotherapy and radiotherapy [50]. The sumoylation of the KAP1 at K554, K779, and K804 attenuates the acetylation and augments the methylation of H3K9 at the p21 promoter, thus repressing the expression of p21 and promoting MCF-7 cell proliferation [137].

While only a few examples of the crosstalk between PTMs in breast cancer have been described here, these examples suggest that we need to consider the interactions between the different PTMs while studying the functions of a certain PTM of any protein.

## Clinical trials on PTMs in breast cancer

**Fig. 8:**
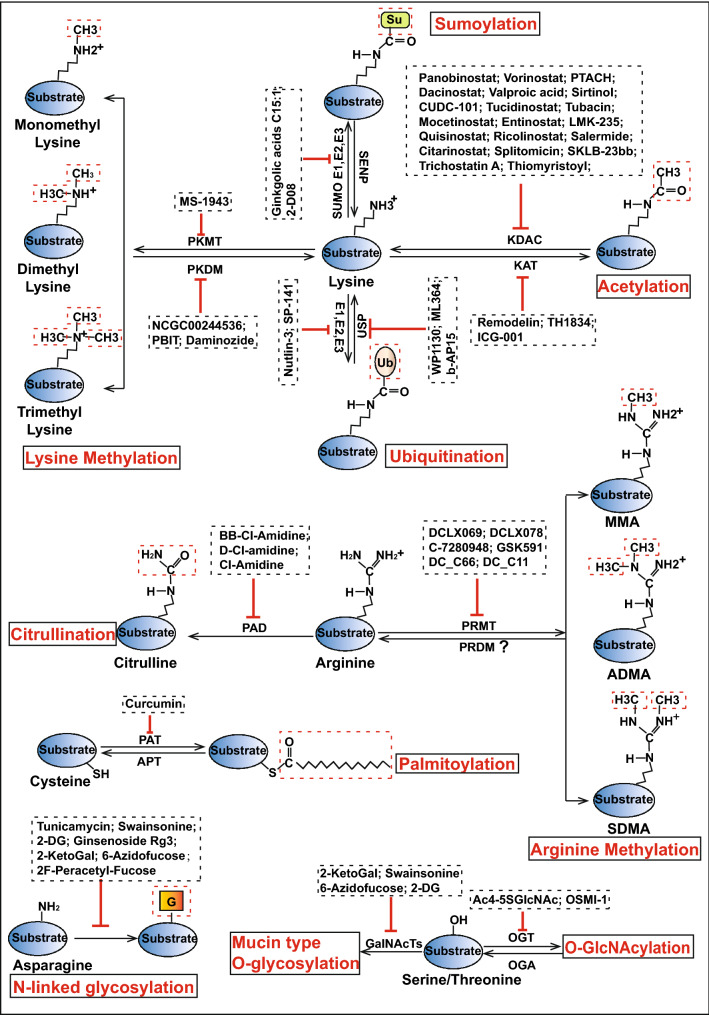
The small molecule inhibitors targeting different PTMs in breast cancer

Based on the mechanisms underlying the PTMs in the oncogenesis and progression of breast cancer, as well as the pre-clinical trials on inhibitors targeting the PTM-associated enzymes for the treatment of breast cancer (Fig. 8; Additional file 1: Table S2), we explored the anti-cancer effects of these inhibitors in breast cancer patients. Several inhibitors targeting protein phosphorylation has been widely studied in clinical trials and some of them, such as Herceptin and gefitinib, are also used clinically [40, 230, 231]. Thus, in the following section, we summarize the PTMs (apart from protein phosphorylation)-associated clinical trials (https://www.clinicaltrials.gov/) that have been carried out worldwide (Additional file 1: Fig. S3). The PTM-associated clinical trials with published articles in breast cancer patients are summarized in Table 1, and those without published articles are summarized in Additional file 1: Table S3.

**Table 1:** The PTM-associated clinical trials with published articles in breast cancer patients

Type of PTMs	Identifiers/Ref	Title	Phase	Diseases	Intervention	Status
Acetylation	NCT01105312 [233]	Panobinostat and Letrozole in Treating Patients With Metastatic Breast Cancer	I/II	Breast Cancer	Drug:letrozole Drug:panobinostat	Completed
	NCT00365599 [237]	Phase II Trial of SAHA & Tamoxifen for Patients With Breast Cancer	II	Breast Cancer	Drug:Vorinostat Drug: Tamoxifen	Completed
	NCT00616967 [254]	Carboplatin and Nab-Paclitaxel With or Without Vorinostat in Treating Women With Newly Diagnosed Operable Breast Cancer	II	Breast Cancer	Drug:carboplatin Drug: paclitaxel albumin-stabilized nanoparticle formulation Drug: vorinostat	Active, not recruiting
	NCT02395627 [241]	Reversing Therapy Resistance With Epigenetic-Immune Modification	II	Breast Neoplasms	Drug: Tamoxifen Drug: Vorinostat Drug:Pembrolizumab	Terminated
	NCT00395655 [236]	Hydralazine and Valproate Added to Chemotherapy for Breast Cancer	II	Locally Advanced Breast Cancer	Drug: Hydralazine Drug: Valproic Acid	Terminated
	NCT02482753 [239]	Trial of Chidamide in Combination With Exemestane in Patients With Advanced Breast Cancer	III	Breast Cancer	Drug: Chidamide Drug: exemestane	Active, not recruiting
	NCT02833155 [235]	Entinostat in Chinese Postmenopausal Women Patients With Locally Recurrent or Metastatic Breast Cancer	I	Breast Cancer	Drug: Entinostat Drug: Exemestane	Completed
	NCT00676663 [238]	Study to Evaluate Exemestane With and Without Entinostat (SNDX-275) in Treatment of Postmenopausal Women With Advanced Breast Cancer	II	Breast Cancer ER + Breast Cancer	Drug: Entinostat Drug: Exemestane	Completed
	NCT04296942 [255]	BN-Brachyury, Entinostat, Adotrastuzumab Emtansine and M7824 in Advanced Stage Breast Cancer (BrEAsT)	I	Breast Cancer Metastatic Breast Cancer	Drug: Entinostat Biological: M7824 Biological: Ado-trastuzumab emtansine	Completed
	NCT01349959 [252]	Azacitidine and Entinostat in Treating Patients With Advanced Breast Cancer	II	Male Breast Carcinoma Recurrent Breast Carcinoma	Drug: Azacitidine Drug: Entinostat	Active, not recruiting
	NCT02115282 [253, 240]	Entinostat in Patients With Recurrent Advanced Hormone Receptor-Positive Breast Cancer	III	Recurrent Breast Carcinoma	Drug: Entinostat Drug: Exemestane Drug: Goserelin Acetate	Active, not recruiting
	NCT02623751 [234]	Study of KHK2375 in Subjects With Advanced or Recurrent Breast Cancer	I	Breast Cancer	Drug: entinostat Drug: Exemestane	Active, not recruiting
Glycosylation	NCT00096707 [232]	Dose Escalation Trial of 2-Deoxy-D-Glucose (2DG) in Subjects With Advanced Solid Tumors	I	Lung Cancer Breast Cancer Pancreatic Cancer Head and Neck Cancer Gastric Cancer	Drug: 2-DG	Completed

However, clinical trials on PTMs in breast cancer are primarily focus on protein acetylation, especially the usage of KDAC inhibitors in breast cancer patients (Table 1, Additional file 1: Table S3). Most of these clinical trials are only in their initial stages and have been successful in confirming the safety of the PTM-inhibitors [232–235], and these trials are primarily focused on the assessment of the therapeutic effects of combination treatment regimens such as PTMs-inhibitors combined with endocrine or chemotherapy [233, 235–240]. For example, a phase I study analyzed the safety, maximum tolerated dose, pharmacokinetics, and bioavailability of oral panobinostat (20 mg, three times weekly) and confirmed that panobinostat can be safely administrated for antitumor activity [233]. Several clinical trials demonstrated the combination therapy with entinostat plus exemestane showed safety and encouraging efficacy in ER positive advanced breast cancer patients [234, 235, 238]. A phase II clinical trial reported that the combination of vorinostat (400 mg/day) and tamoxifen reverse hormone resistance in patients with ER-positive metastatic breast cancer [237]. Another phase III clinical trial proved that tucidinostat plus exemestane improved median progression-free survival (from 3.8 months to 7.4 months) compared with placebo group in hormone receptor-positive, HER2-negative breast cancer patients [239]. In addition, a phase II trial showed that PD-L1 negative ER positive breast cancer patients may benefit from immune checkpoint inhibitor combined with KDAC inhibitor therapy [241]. Moreover, a phase I study confirmed the safety and efficacy of 2-DG (63 mg/kg/day) combined with docetaxel in advanced solid tumors [232].

Although many inhibitors of PTMs were initially researched on histone modifications, there is no doubt that they also have corresponding regulatory effects on non-histone proteins in view of enzymatic activity, so when these drugs are applied to the systemic treatment of breast cancer patients, it is inevitable that these drugs also play a role in non-histone proteins. Therefore, the drugs used in these clinical trials may act through the regulation of histones and non-histone proteins at the same time. The specific mechanism needs to be further studied.

## Conclusion and perspectives

Breast cancer is a highly heterogeneous disease. Studies mainly focus on the molecular mechanisms at the genome and protein levels without taking into account the protein PTMs might be insufficient for the appropriate treatment of breast cancer. The recent advances in omics technologies [14, 16, 20, 29] such as mass spectrometry, high-throughput sequencing, and bioinformatics, have made the identification of PTMs and their underlying mechanisms regulating breast cancer tumorigenesis and progression possible. Here, we discussed the various studies analyzing the underlying mechanisms of protein PTMs that regulate breast cancer, and thus demonstrated the significance of PTMs, broadened our understanding of the relationship between PTMs and breast cancer, and provided a new perspective in breast cancer treatment. However, future studies are required to address several important questions that are still unanswered.

Some of the components in PTMs, such as the erasers of arginine methylation, the target proteins and modified sites of writers, and the readers of novel acylation, have not been identified and need to be further studied. Moreover, considering the extensive number of PTMs in breast cancer, further studies are crucial to elucidate the underlying molecular mechanisms and address questions. Whether the activation of all these PTMs is similar to that of phosphorylation by multiple stimuli such as hypoxia, inflammatory factors, growth factors, cytokines, and DNA damage and whether these PTMs are mediated via signal cascade amplification like phosphorylation are needed to be explored [33–35, 242, 243]. Acylation refers to the modification of lysine residues by acyl molecules with different chemical structures [53]. Recent studies have reported novel acylations that takes place in histone, such as lactylation, propionylation, butyrylation, succinylation, and crotonylation [53, 224]. For example, histone lactylation promotes the transcription of YTHDF2 (the m^6^A reader protein) to participate in the progression of ocular melanoma [244]. Whether these novel acylation modifications also occur in the development and progression of breast cancer, and whether there is a crosstalk between these novel acylation modifications and currently known PTMs, are needed to be addressed in the future studies. Moreover, the mechanisms of ADP-ribosylation involving in the regulation of breast cancer [245, 246] and its relationship with other glycosylation modifications awaits further study. Furthermore, a single enzyme may have opposite effects on promoting or inhibiting tumor progression. However, how these modifications that occur at multiple sites exactly work together, and the biological consequences of the different multi-site PTMs, are still unknown. Therefore, due to the complexity of the PTM network, it might be difficult to speculate the effects of any modification merely by the enzymes and target protein. Little is known about how PTMs coordinately regulate different proteins involved in a specific biological process. In addition, breast cancer is a heterogeneous disease, but in addition to the PTMs occurring in ER, PR, and HER2 proteins are subtype-specific in breast cancer [106, 165, 171, 177, 196, 247–249], most PTMs occurring on oncoproteins or tumor suppressor proteins, such as Pin1 and p53, are non-selective in any subtype of breast cancer [65, 170, 178–180, 250, 251]. Therefore, specific PTMs of different breast cancer subtypes deserve further research. Intriguingly, a variety of PTMs, such as acetylation, glycosylation, sumoylation, methylation, and ubiquitination, occur on the ERα [64, 102, 171, 177, 196, 247, 248], which attracts our attention, so it is of great significance to elucidate the protein post-translational modification regulatory network of ERα for the treatment of ER positive breast cancer patients.

Many inhibitors targeting PTMs in breast cancer have now been developed and are under preclinical trials and different phases of clinical trials [232–241, 252–255]. These inhibitors might have promising applications in the personalized treatment of breast cancer. For example, in ER positive subtype; the combination of pan-HDAC inhibitor vorinostat (400 mg/day) and tamoxifen reverse hormone resistance in patients with ER-positive metastatic breast cancer [237]. Moreover, the combination therapy with entinostat plus exemestane showed safety and encouraging efficacy in ER positive advanced breast cancer patients [234, 235, 238]. In HER2 positive subtype; pan-sirtuin inhibitor, sirtinol, increases the sensitivity of lapatinib in anti-HER2 targeted treatment in breast cancer [70]. Class I and II HDAC inhibitor, valproic acid, may disrupt the functions of Hsp90, leading to the downregulation of its client protein HER2 [77]. Tunicamycin, which inhibits the generation of long-chain terpene testosterol diphosphate that is required for *N*-glycosylation. HER2 protein has seven *N*-glycosylation sites, whose glycosylation can activate the MAPK signaling pathway to promote breast cancer progression, the reduction in cell surface glycosylation together with the increased sensitivity of HER2 positive cells to Herceptin and doxorubicin has been found following tunicamycin treatment [106]. In TNBC subtype which has no targeted treatments currently, class I/ II HDAC inhibitor, trichostatin A, can induce the re-expression of ERα in ER-negative MDA-MB-231 cells, thus suggesting that KDAC inhibition may be a potential therapeutic strategy for TNBC [256]. The small-molecule pharmacological OGT inhibitors OSMI-1 is efficient in inducing cell death and growth inhibition in TNBC cells by the enhanced proteasomal degradation of hairy and enhancer of split-1 (HES1), that interact with the Fanconi-anemia complex to accelerate DNA damage repair [257]. The STM108 antibody can specifically recognize the Asn192 and Asn200 glycosylation sites of PD-L1 and the antibody–drug conjugate, STM108-ADC, induces potent drug-induced cytotoxic activities and bystander effects to kill TNBC cells both in vivo and in vitro (Fig. 4B) [123]. This implies that targeting protein glycosylation may be a potential means to enhance the effects of immune checkpoint therapy in TNBC. However, we are still far from clinically applying therapies targeting PTMs for the treatment of breast cancer. Our understanding of the underlying mechanisms of action of the currently known PTMs is far from complete, for instance, whether there is a change in the PTMs throughout breast cancer anti-tumor treatment is still unclear. Further, many of these agents working on both histones and non-histones, lack specificity, this may account for the higher adverse effects in the PTMs inhibitor group than placebo group in clinical trials [238–240]. Therefore, some inhibitors with higher specificity have yet to be developed. Aberrant PTMs may reduce the sensitivity of tumor cells to anti-tumor therapy. Some previous studies in animal models and tumor cell lines have shown that some small-molecule inhibitors promote cancer cell death when combined with anti-tumor therapy. However, further studies are required to confirm these effects in humans.

In conclusion, unconventional PTMs act as the helmsmen regulating the oncogenesis and progression of breast cancer and may potentially be used in breast cancer treatment. We believe that a deeper understanding of PTMs may facilitate a breakthrough in conventional breast cancer treatment and greatly help breast cancer patients.

## Supplementary Information

**Additional file 1: Table S1.** Potential mechanisms of PTM-associated enzymes involved in breast cancer oncogenesis and progression. **Table S2.** Inhibitors targeting PTM-associated enzymes to regulate breast cancer progression. **Table S3.** The PTM-associated clinical trials without published articles in breast cancer patients. **Fig. S1.** Different glycosylation modification in breast cancer. **Fig. S2.** The mechanisms underlying protein ubiquitination. **Fig. S3.** The distribution of protein PTM-associated clinical trials worldwide

## Data Availability

Not applicable.

## Abbreviations

AIB1: Amplified in breast cancer 1
MOF: Males absent on the first protein
KATs: Lysine acetyltransferases
K: Lysine
IL-8: Interleukin-8
NF-κB: Nuclear factor-kappa B
HDAC1: Histone deacetylase 1
ERα: Estrogen receptor α
DBD: DNA binding domain
AF-2: Transcription activation function domain 2
KDACs: Lysine deacetylases
HSPA5: Heat-shock protein 5
PERK: Protein kinase RNA-like endoplasmic reticulum kinase
UPR: Unfolded protein response
CBP: CREB-binding protein
STAT3: Signal transducer and activator of transcription 3
EMT: Epithelial-mesenchymal transition
BRD4: Bromodomain-containing protein 4
SIRT2: Sirtuin 2
CSCs: Cancer stem cells
ALDH1A1: Aldehyde dehydrogenase A1
PCAF: P300/CBP-associated factor
ATAT1: α-Tubulin *N*-acetyltransferase 1
SOD2: Superoxide dismutase2
NAT10: *N*-Acetyltransferase 10
MORC2: Microrchidia family CW-type zinc finger 2
FOXO3: Forkhead Box O3
CCL18: C–C motif chemokine 18
ACAP4: ARF6 GTPase-activating protein4
HOXB13: Homeobox B13
IL-6: Interleukin-6
TAM: Tamoxifen
TIP60: 60 KDa Tat-interactive protein
GT: Glycosyltransferases
OGT: O-GlcNAc transferase
Ser: Serine
GalNAc-T4: *N*-Acetylgalactosaminyltransferase4
FOXA1: Forkhead box protein A1
MUC1: Mucin 1
FUT4: Fucosyltransferases 4
TβR: Transforming growth factor-β(TGF-β) serine/threonine kinase receptor
HER2: Human epidermal growth factor receptor 2
MAPK: Mitogen-activated protein kinase
RPN2: Ribophorin II
MDR1: Multidrug resistance protein 1
Asn: Asparagine
EpCAM: Epithelial cell adhesion molecule
B3GNT3: β-1,3-*N*-Acetylglucosaminyl transferase
C/EBPbeta1: CCAAT/enhancer binding protein beta1
FAs: Focal adhesions
PR: Progesterone receptor
SRC-1: Steroid receptor coactivator 1
SENPs: SUMO-specific proteases
Pin1: Protein interacting with NIMA (never in mitosis A)-1
KAP1: KRAB domain-associated protein 1
PRMTs: Protein lysine methyltransferases
BAF155: BRG1-associated factor 155
CARM1: Co-activator associated arginine methyltransferase 1
SET6: SET-domain-containing protein methyltransferase 6
WDR5: WD repeat domain 5
PKDMs: Protein lysine demethylases
PADs: Peptidylarginine deiminases
RNAP2: RNA polymerase II
PADI2: Peptidyl arginine deiminase 2
GSK3β: Glycogen synthase kinase-3β
RARRES3: Retinoic acid receptor responder3
LRP6: Lipoprotein receptor-related protein 6
ITGβ4: Integrin β4
PFK1: Phosphofructokinase 1
PPP: Pentose phosphate pathway
MMP9: Matrix metalloproteinase-9
RNF31: Ring finger protein 31
RBR: RING-in-between-RING
RBCK1: Ran Bp-type and C3HC4-type zinc finger-containing protein 1
NEMO: Inhibitor of κB Kinase (IKK) γ
NHEJ: Non-homologous end joining
DNA-PKcs: DNA-dependent protein kinase catalytic subunit
PARP1: Poly(ADP-ribose) polymerase 1
BER: Base excision repair
PARK2: Parkinson protein 2
