# Supplementary material for: Unconventional protein post-translational modifications: the helmsmen in breast cancer
Source: Cell Biosci. 2022 Feb 25;12:22. doi: 10.1186/s13578-022-00756-z (PMC8881842; doi:10.1186/s13578-022-00756-z)
Supplement: Supplementary file 1 — Additional file 1: Table S1. Potential mechanisms of PTM-associated enzymes involved in breast cancer oncogenesis and progression. Table S2. Inhibitors targeting PTM-associated enzymes to regulate breast cancer progression. Table S3. The PTM-associated clinical trials without published articles in breast cancer patients. Fig. S1. Different glycosylation modification in breast cancer. Fig. S2. The mechanisms underlying protein ubiquitination. Fig. S3. The distribution of protein PTM-associated clinical trials worldwide [file 13578_2022_756_MOESM1_ESM.docx]

**Supplementary Materials**

**Table S1.** **Potential mechanisms of PTM-associated enzymes involved in breast cancer oncogenesis and progression**

| Type of PTMs | Enzymes | Species | Target proteins | Mode | Function | Mechanism | Ref. |
| --- | --- | --- | --- | --- | --- | --- | --- |
| Acetylation | MOF | KATs | AIB1  (K276) | Oncogenic potential | ↑ proliferation | Activate E2F1 to promote cancer cell proliferation | [1] |
|  | P300 | KATs | RelA/p65  (K218/221/  310) | Oncogenic potential | ↑ angiogenesis ↑ metastasis | Enhance the transcription of IL-8 via NF-κB pathway | [2] |
|  |  |  | HSPA5  (K353) | Oncogenic potential | ↑ metastasis | Inhibit its  ubiquitination degradation | [3] |
|  |  |  | HOXB13  (K277) | Oncogenic potential | ↑ TAM resistance | Inhibit ERα expression and upregulate IL-6 expression | [4] |
|  | NAT10 | KATs | MORC2  (K767) | Oncogenic potential | ↑ radiotherapy  resistance and chemotherapy resistance | Regulate DNA damage-induced G2 checkpoint arrest;  decrease sensitivity to DNA-damaging  therapeutic agents | [5] |
|  | PCAF | KATs | ACAP4  (K311) | Oncogenic potential | ↑ migration  ↑invasion | Promote CCL 18 elicited cell migration | [6] |
|  | HDAC1 | KDACs | ERα | Oncogenic potential | ↑proliferation | Direct Interaction with DBD and AF-2 domains of ERα | [7] |
|  | Tip60 | KATs | Twist  (K73/K76) | Oncogenic potential | ↑ EMT | Promote its interact with BRD4 to promote the expression of WNT5A and activate WNT pathway | [8] |
|  | SIRT2 | KDACs | ALDH1A1  (K353) | Oncogenic potential | ↑ breast CSCs | Promote ALDH1A1  enzymatic activity and CSCs self- renewal | [9] |
|  | HDAC6 | KDACs | Survivin  (K129) | Oncogenic potential | ↓ apoptotic | Promote survivin nuclear export to blocks programmed cell death | [10] |
|  |  |  | α-tubulin  (K40) | Oncogenic potential | ↑ migration ↑invasion | Inhibit a stable microtubule;  promote gelatin degradation | [11-13] |
|  |  |  | cortactin | Oncogenic potential | ↑ migration ↑invasion | Increase invadopodia formation and  increase its binding to F-actin | [11, 12] |
|  |  |  | HSPA5  (K118/122/123/125/138/152/154/352/353/376/633) | Oncogenic potential | ↓cell death | Reduce HSPA5 acetylation and increase its binding to PERK to inhibit lethal UPR | [14] |
|  |  |  | HSPA5  (K353) | Tumor suppressor  potential | ↓metastasis | Deacetylation of HSPA5 promote its  ubiquitination degradation | [3, 15] |
|  | SIRT3 | KDACs | SOD2  (K68) | Tumor suppressor  potential | ↓ CSCs | Inhibit mitochondrial reactive oxygen species and HIF2α-  dependent CSCs reprogramming | [16] |
|  | CBP | KATs | Survivin  (K129) | Tumor suppressor  potential | ↓proliferation | Maintains survivin nuclear localization to inhibit STAT3 transactivation | [10, 17] |
|  | PCAF | KATs | ALDH1A1  (K353) | Tumor suppressor  potential | ↓ CSCs | Inhibit ALDH1A1  enzymatic activity and the population of CSCs | [9] |
|  | ATAT1 | KATs | α-tubulin  (K40) | Tumor suppressor  potential | ↓ invasion | Sustain a stable microtubule structure | [12] |
|  | P300 | KATs | FOXO3  (K242/245) | Tumor suppressor  potential | ↓ proliferation  ↑Lapatinib sensitivity | promote the  nuclear translocation and activation of FOXO3 | [18] |
|  | Tip60 | KATs | cortactin | Tumor suppressor  potential | ↓migration ↓invasion | Suppresses invadopodia formation | [19] |
| Glycosylation | OGT | GT | Cofilin  (S108) | Oncogenic potential | ↑ invasion | Promote actin dynamics and proper localization of invadopodia | [20] |
|  |  |  | vimentin | Oncogenic potential | ↑ invasion | Promote its expression and cell migration | [21] |
|  |  |  | PFK1  (S529) | Oncogenic potential | ↑proliferation ↑tumor formation | Suppress its activity and redirect glucose flux through the PPP, promote protein O-GlcNAcylation | [22] |
|  |  |  | Snail1  (S112) | Oncogenic potential | ↑ EMT | Inhibit its ubiquitin-dependent degradation and the transcription of E-cadherin | [23] |
|  |  |  | PR | Oncogenic potential | ↑proliferation | promote transcription activity of PR-target genes | [24] |
|  |  |  | β-catenin,p120 | Oncogenic potential | ↑EMT | Decrease the membrane translocation of E-cadherin | [25] |
|  | GalNAc-T4 | GT  (GalNAc-Ts) | FOXA1 | Oncogenic potential | ↑ proliferation | Promote ERα recruitment and cyclin D1 expression | [26] |
|  | GalNAc-T6 | GT  (GalNAc-Ts) | ERα  (S573) | Oncogenic potential | ↑cell viability | Promote its nuclear localization and downstream target transcription | [27] |
|  |  |  | MUC1 | Oncogenic potential | ↑proliferation  ↑invasion | Sustain MUC1 stability and reduce cell adhesion | [28, 29] |
|  | FUT8 | GT  (FUT) | TβRI/II | Oncogenic potential | ↑ EMT  ↑invasion | Activate the TGF-β signaling pathway | [30] |
|  | GT | GT | HER2  (N68/124/187/259/530/571/  548) | Oncogenic potential | ↑ treatment resistance | Active the MAPK signaling pathway | [31] |
|  |  |  | EpCAM  (N74/111/198) | Oncogenic potential | ↑ migration  ↑ invasion | Upregulate the expression of fibronectin and integrin β1, and activate FAK-PI3K-Akt pathway | [32, 33] |
|  | B3GNT3 | GT | PD-L1  (N192/200) | Oncogenic potential | ↑ immune suppression | Promote its interaction with PD-1 in TNBC | [34-36] |
|  | RPN2 | GT | CD63 | Oncogenic potential | ↑invasion and chemotherapy resistance invasion | Promote CD63 interact with MDR1 and co-localized in cell membrane | [37, 38] |
|  |  |  | MDR1 | Oncogenic potential | ↑drug resistance | Reduce cancer cell apoptosis induced by docetaxel | [37] |
| Sumoylation | SUMO-  Enzyme complex | SUMO-  Enzyme complex | C/EBPbet-a1  (K173) | Oncogenic potential | ↓senescence | Suppressed its ability to facilitate oncogene induced senescence in breast cancer cells | [39] |
|  |  |  | BRCA1  (K32/1690) | Oncogenic potential | ↑proliferation | Induce G0/G1 phase transition in MCF-7 cells | [40] |
|  |  |  | Talin  (K2445/841) | Oncogenic potential | ↑migration | Regulate FAs disassemble and link cytoskeleton to extracellular matrix | [41] |
|  |  |  | PR  (K388) | Oncogenic potential | ↑proliferation | Promote its interaction with SRC-1 and PR signal transduction | [42] |
|  |  |  | SRC-1  (K732/774) | Oncogenic potential | ↑proliferation | Promote PR downstream target genes transcription | [42] |
|  |  |  | FOXM1B  (K463) | Oncogenic potential | ↑proliferation  ↑ migration | Inhibit the expression of p21;  promote the expression of JNK1 | [43] |
|  |  |  | TβRI  (K389) | Oncogenic potential | ↑metastasis | Activate TGFβ-Smad4 pathway. | [44] |
|  |  |  | KAP1  (K554/779/  804) | Oncogenic potential | ↑ proliferation | Augment the methylation of H3K9 at the p21 promoter to repress the expression of p21 | [45] |
|  | SENP1 | SENPs | Pin1  (K6/63) | Oncogenic potential | ↑ proliferation | Activate oncogenes and inhibit tumor suppressor genes | [46-48] |
|  | SENP2 |  | Smad4  (K159) | Oncogenic potential | ↑ EMT ↑migration | Active TGFβ-Smad4 signaling pathway | [49] |
|  | SENP5 |  | TβRI | Oncogenic potential | ↑metastasis | Increase the expression of TβRI and downstream MMP9 transcription | [50] |
|  | SUMO-  Enzyme complex | SUMO-  Enzyme complex | Smad4  (K159) | Tumor suppressor  potential | ↓EMT ↓migration | Promote the interaction between smad4 and Daxx, thereby inhibit the transcriptional activity of samd4 | [49, 51] |
|  |  |  | Pin1  (K6/63) | Tumor suppressor  potential | ↓proliferation | Suppress its activity and oncogenic function | [47] |
|  | SENP2 | SENPs | ERα | Tumor suppressor  potential | ↓proliferation | Recruit HDAC3 to the promoter of ERα  and inhibit the G1/S transition of MCF-7 cells | [52] |
|  |  |  | FOXM1B  (K463) | Tumor suppressor  potential | ↓proliferation  ↓migration | Activate its target genes miR200 and p21, increase the expression of E-cadherin | [43] |
| Methylation | PRMT7 | PRMTs | PRMT7  (R531) | Oncogenic potential | ↑ EMT | Repress the expression of E-cadherin | [53] |
|  | PRMT5 |  | PDCD4  (R110) | Oncogenic potential | ↑tumor growth | Co-expression of PDCD4 and PRMT5 promote tumor growth | [54] |
|  |  |  | AKT  (R391) | Oncogenic potential | ↑proliferation | Activate PI3K-AKT pathway | [55] |
|  |  |  | P53  (R333/335/  337) | Oncogenic potential | ↑tumor growth | Inhibit cancer cell apoptosis in response to double-stranded DNA damage | [56] |
|  | CARM1 |  | BAF155  (R1064) | Oncogenic potential | ↑migration ↑metastasis | Regulate the c-Myc signaling pathway | [57] |
|  | PRMT1 |  | ERα  (R260) | Oncogenic potential | ↑proliferation ↓apoptotic | Trigger downstream PI3K-Src-Akt signaling pathway | [58] |
|  | SET6 | PKMTs | WDR5  (K207/325) | Oncogenic potential | ↑proliferation ↑ metastasis | Maintain global histone tri-methylation of lysine 4 on histone H3  levels | [59] |
|  | SMYD2 |  | P53  (K370) | Oncogenic potential | ↑tumor growth | Inhibit its antitumor ability | [60, 61] |
|  | LSD1 | PKDMs | ERα  (K266) | Oncogenic potential | ↑tumor growth | Promote ERα target genes expression | [62] |
|  | KDM2A |  | TET2 | Oncogenic potential | ↑EMT  ↑angiogenesis | Inhibit the expression of E-cadherin  activate the Notch signaling pathway | [63, 64] |
|  | JMJD6 | PRDMs | ERα  (K260) | Tumor suppressor  potential | ↓proliferation | Suppress the activating of PI3K-Src-Akt signal pathway | [58, 65] |
|  | SMYD2 | PKMTs | ERα  (K266) | Tumor suppressor  potential | ↓ proliferation | Attenuate the chromatin recruitment of ERα and its target genes expression | [62] |
| Ubiquitination | RNF31 | RBR E3 ligase | ERα | Oncogenic potential | ↓proliferation | Promote the transcription of ERα signal downstream oncogenic proteins | [66, 67] |
|  | RNF31, RBCK1 |  | NEMO | Oncogenic potential | ↓apoptosis | Activate the NF-κB signaling pathway | [67, 68] |
|  | PARK2 |  | HIF1α  (K477) | Tumor suppressor  potential | ↓metastasis | Promote HIF1α ubiquitination and degradation | [69] |
|  | RNF144A |  | PARP1 | Tumor suppressor  potential | ↑apoptosis | Inhibit PARP1 induced BER pathway | [70] |
|  | RNF144A |  | DNA-PKcs | Tumor suppressor  potential | ↑ apoptosis  ↑autophagy | Inhibit DNA-PKcs induced NHEJ pathway | [71] |
| Citrullination | PADI2 | PADs | RNAP2  (R1810) | Oncogenic potential | ↑proliferation | Promote gene transcription | [72, 73] |
|  | PADI2 |  | Rho family | Oncogenic potential | ↓migration | Regulate the EGF-PI3K signaling pathway | [74] |
|  | PAD4 |  | GSK3β  (R3) | Tumor suppressor  potential | ↓ EMT | Increase its nuclear  Localization and inhibit TGF-β signal pathway | [75] |
| Palmitoylation | RARRES3 | APT | LRP6 | Tumor suppressor  potential | ↓proliferation↓ EMT | Inhibit Wnt/β-catenin pathway | [76] |
|  | PAT | PAT | CD44  (C286/295) | Tumor suppressor  potential | ↓migration | Increase CD44 raft affiliation and decrease CD44-ezrin interaction | [77] |
|  | DHHC3 |  | ITGβ4  (C732/736/738/739/742) | Oncogenic potential | ↑ migration | Mediates its trafficking and signaling competency | [78-80] |

Note: ↑, promote; ↓, inhibit

**References**

1.You D, Zhao H, Wang Y, Jiao Y, Lu M,Yan S. Acetylation Enhances the Promoting Role of AIB1 in Breast Cancer Cell Proliferation. Mol Cells. 2016;39(9):663-8.

2.Malonia SK, Yadav B, Sinha S, Lazennec G,Chattopadhyay S. Chromatin remodeling protein SMAR1 regulates NF-kappaB dependent Interleukin-8 transcription in breast cancer. Int J Biochem Cell Biol. 2014;55220-6.

3.Chang YW, Chen HA, Tseng CF, Hong CC, Ma JT, Hung MC, et al. De-acetylation and degradation of HSPA5 is critical for E1A metastasis suppression in breast cancer cells. Oncotarget. 2014;5(21):10558-70.

4.Liu B, Wang T, Wang H, Zhang L, Xu F, Fang R, et al. Oncoprotein HBXIP enhances HOXB13 acetylation and co-activates HOXB13 to confer tamoxifen resistance in breast cancer. J Hematol Oncol. 2018;11(1):26.

5.Liu HY, Liu YY, Yang F, Zhang L, Zhang FL, Hu X, et al. Acetylation of MORC2 by NAT10 regulates cell-cycle checkpoint control and resistance to DNA-damaging chemotherapy and radiotherapy in breast cancer. Nucleic Acids Res. 2020;48(7):3638-3656.

6.Song X, Liu W, Yuan X, Jiang J, Wang W, Mullen M, et al. Acetylation of ACAP4 regulates CCL18-elicited breast cancer cell migration and invasion. J Mol Cell Biol. 2018;10(6):559-572.

7.Kawai H, Li H, Avraham S, Jiang S,Avraham HK. Overexpression of histone deacetylase HDAC1 modulates breast cancer progression by negative regulation of estrogen receptor alpha. Int J Cancer. 2003;107(3):353-8.

8.Shi J, Wang Y, Zeng L, Wu Y, Deng J, Zhang Q, et al. Disrupting the interaction of BRD4 with diacetylated Twist suppresses tumorigenesis in basal-like breast cancer. Cancer Cell. 2014;25(2):210-25.

9.Zhao D, Mo Y, Li MT, Zou SW, Cheng ZL, Sun YP, et al. NOTCH-induced aldehyde dehydrogenase 1A1 deacetylation promotes breast cancer stem cells. J Clin Invest. 2014;124(12):5453-65.

10.Riolo MT, Cooper ZA, Holloway MP, Cheng Y, Bianchi C, Yakirevich E, et al. Histone deacetylase 6 (HDAC6) deacetylates survivin for its nuclear export in breast cancer. J Biol Chem. 2012;287(14):10885-93.

11.Rey M, Irondelle M, Waharte F, Lizarraga F,Chavrier P. HDAC6 is required for invadopodia activity and invasion by breast tumor cells. Eur J Cell Biol. 2011;90(2-3):128-35.

12.Castro-Castro A, Janke C, Montagnac G, Paul-Gilloteaux P,Chavrier P. ATAT1/MEC-17 acetyltransferase and HDAC6 deacetylase control a balance of acetylation of alpha-tubulin and cortactin and regulate MT1-MMP trafficking and breast tumor cell invasion. Eur J Cell Biol. 2012;91(11-12):950-60.

13.Hubbert C, Guardiola A, Shao R, Kawaguchi Y, Ito A, Nixon A, et al. HDAC6 is a microtubule-associated deacetylase. Nature. 2002;417(6887):455-8.

14.Rao R, Nalluri S, Kolhe R, Yang Y, Fiskus W, Chen J, et al. Treatment with panobinostat induces glucose-regulated protein 78 acetylation and endoplasmic reticulum stress in breast cancer cells. Mol Cancer Ther. 2010;9(4):942-52.

15.Chang YW, Tseng CF, Wang MY, Chang WC, Lee CC, Chen LT, et al. Deacetylation of HSPA5 by HDAC6 leads to GP78-mediated HSPA5 ubiquitination at K447 and suppresses metastasis of breast cancer. Oncogene. 2016;35(12):1517-28.

16.He C, Danes JM, Hart PC, Zhu Y, Huang Y, de Abreu AL, et al. SOD2 acetylation on lysine 68 promotes stem cell reprogramming in breast cancer. Proc Natl Acad Sci U S A. 2019;116(47):23534-23541.

17.Wang H, Holloway MP, Ma L, Cooper ZA, Riolo M, Samkari A, et al. Acetylation directs survivin nuclear localization to repress STAT3 oncogenic activity. J Biol Chem. 2010;285(46):36129-37.

18.Mahmud Z, Gomes AR, Lee HJ, Aimjongjun S, Jiramongkol Y, Yao S, et al. EP300 and SIRT1/6 Co-Regulate Lapatinib Sensitivity Via Modulating FOXO3-Acetylation and Activity in Breast Cancer. Cancers (Basel). 2019;11(8):

19.Sun Y, Sun J, Lungchukiet P, Quarni W, Yang S, Zhang X, et al. Fe65 Suppresses Breast Cancer Cell Migration and Invasion through Tip60 Mediated Cortactin Acetylation. Sci Rep. 2015;511529.

20.Huang X, Pan Q, Sun D, Chen W, Shen A, Huang M, et al. O-GlcNAcylation of cofilin promotes breast cancer cell invasion. J Biol Chem. 2013;288(51):36418-25.

21.Komura K, Ise H,Akaike T. Dynamic behaviors of vimentin induced by interaction with GlcNAc molecules. Glycobiology. 2012;22(12):1741-59.

22.Yi W, Clark PM, Mason DE, Keenan MC, Hill C, Goddard WA, 3rd, et al. Phosphofructokinase 1 glycosylation regulates cell growth and metabolism. Science. 2012;337(6097):975-80.

23.Park SY, Kim HS, Kim NH, Ji S, Cha SY, Kang JG, et al. Snail1 is stabilized by O-GlcNAc modification in hyperglycaemic condition. EMBO J. 2010;29(22):3787-96.

24.Trinca GM, Goodman ML, Papachristou EK, D'Santos CS, Chalise P, Madan R, et al. O-GlcNAc-Dependent Regulation of Progesterone Receptor Function in Breast Cancer. Horm Cancer. 2018;9(1):12-21.

25.Gu Y, Mi W, Ge Y, Liu H, Fan Q, Han C, et al. GlcNAcylation plays an essential role in breast cancer metastasis. Cancer Res. 2010;70(15):6344-51.

26.Niang B, Jin L, Chen X, Guo X, Zhang H, Wu Q, et al. GalNAc-T4 putatively modulates the estrogen regulatory network through FOXA1 glycosylation in human breast cancer cells. Mol Cell Biochem. 2016;411(1-2):393-402.

27.Deng B, Tarhan YE, Ueda K, Ren L, Katagiri T, Park JH, et al. Critical Role of Estrogen Receptor Alpha O-Glycosylation by N-Acetylgalactosaminyltransferase 6 (GALNT6) in Its Nuclear Localization in Breast Cancer Cells. Neoplasia. 2018;20(10):1038-1044.

28.Ciborowski P,Finn OJ. Non-glycosylated tandem repeats of MUC1 facilitate attachment of breast tumor cells to normal human lung tissue and immobilized extracellular matrix proteins (ECM) in vitro: potential role in metastasis. Clin Exp Metastasis. 2002;19(4):339-45.

29.Park JH, Nishidate T, Kijima K, Ohashi T, Takegawa K, Fujikane T, et al. Critical roles of mucin 1 glycosylation by transactivated polypeptide N-acetylgalactosaminyltransferase 6 in mammary carcinogenesis. Cancer Res. 2010;70(7):2759-69.

30.Tu CF, Wu MY, Lin YC, Kannagi R,Yang RB. FUT8 promotes breast cancer cell invasiveness by remodeling TGF-beta receptor core fucosylation. Breast Cancer Res. 2017;19(1):111.

31.Peiris D, Spector AF, Lomax-Browne H, Azimi T, Ramesh B, Loizidou M, et al. Cellular glycosylation affects Herceptin binding and sensitivity of breast cancer cells to doxorubicin and growth factors. Sci Rep. 2017;743006.

32.Liu X, Gao J, Sun Y, Zhang D, Liu T, Yan Q, et al. Mutation of N-linked glycosylation in EpCAM affected cell adhesion in breast cancer cells. Biol Chem. 2017;398(10):1119-1126.

33.Zhang D, Yang L, Liu X, Gao J, Liu T, Yan Q, et al. Hypoxia modulates stem cell properties and induces EMT through N-glycosylation of EpCAM in breast cancer cells. J Cell Physiol. 2020;235(4):3626-3633.

34.Li CW, Lim SO, Chung EM, Kim YS, Park AH, Yao J, et al. Eradication of Triple-Negative Breast Cancer Cells by Targeting Glycosylated PD-L1. Cancer Cell. 2018;33(2):187-201 e10.

35.Salatino M, Girotti MR,Rabinovich GA. Glycans Pave the Way for Immunotherapy in Triple-Negative Breast Cancer. Cancer Cell. 2018;33(2):155-157.

36.Scott DA,Drake RR. Glycosylation and its implications in breast cancer. Expert Rev Proteomics. 2019;16(8):665-680.

37.Honma K, Iwao-Koizumi K, Takeshita F, Yamamoto Y, Yoshida T, Nishio K, et al. RPN2 gene confers docetaxel resistance in breast cancer. Nat Med. 2008;14(9):939-48.

38.Tominaga N, Hagiwara K, Kosaka N, Honma K, Nakagama H,Ochiya T. RPN2-mediated glycosylation of tetraspanin CD63 regulates breast cancer cell malignancy. Mol Cancer. 2014;13134.

39.Yang P-C, Atwood AA, Jerrell R,Sealy L. Negative Regulation of C/EBPbeta1 by Sumoylation in Breast Cancer Cells. PLoS ONE. 2011;6(9):

40.Vialter A, Vincent A, Demidem A, Morvan D, Stepien G, Venezia ND, et al. Cell cycle-dependent conjugation of endogenous BRCA1 protein with SUMO-2/3. Biochim Biophys Acta. 2011;1810(4):432-8.

41.Huang Z, Barker D, Gibbins JM,Dash PR. Talin is a substrate for SUMOylation in migrating cancer cells. Experimental Cell Research. 2018;370(2):417-425.

42.Chauchereau A, Amazit L, Quesne M, Guiochon-Mantel A,Milgrom E. Sumoylation of the progesterone receptor and of the steroid receptor coactivator SRC-1. J Biol Chem. 2003;278(14):12335-43.

43.Wang CM, Liu R, Wang L, Nascimento L, Brennan VC,Yang WH. SUMOylation of FOXM1B alters its transcriptional activity on regulation of MiR-200 family and JNK1 in MCF7 human breast cancer cells. Int J Mol Sci. 2014;15(6):10233-51.

44.Kang JS, Saunier EF, Akhurst RJ,Derynck R. The type I TGF-beta receptor is covalently modified and regulated by sumoylation. Nat Cell Biol. 2008;10(6):654-64.

45.Lee YK, Thomas SN, Yang AJ,Ann DK. Doxorubicin down-regulates Kruppel-associated box domain-associated protein 1 sumoylation that relieves its transcription repression on p21WAF1/CIP1 in breast cancer MCF-7 cells. J Biol Chem. 2007;282(3):1595-606.

46.Lu KP,Zhou XZ. The prolyl isomerase PIN1: a pivotal new twist in phosphorylation signalling and disease. Nat Rev Mol Cell Biol. 2007;8(11):904-16.

47.Chen CH, Chang CC, Lee TH, Luo M, Huang P, Liao PH, et al. SENP1 deSUMOylates and regulates Pin1 protein activity and cellular function. Cancer Res. 2013;73(13):3951-62.

48.Chen Y, Wu YR, Yang HY, Li XZ, Jie MM, Hu CJ, et al. Prolyl isomerase Pin1: a promoter of cancer and a target for therapy. Cell Death Dis. 2018;9(9):883.

49.Chang CC, Huang YS, Lin YM, Lin CJ, Jeng JC, Liu SM, et al. The role of sentrin-specific protease 2 substrate recognition in TGF-beta-induced tumorigenesis. Sci Rep. 2018;8(1):9786.

50.Cashman R, Cohen H, Ben-Hamo R, Zilberberg A,Efroni S. SENP5 mediates breast cancer invasion via a TGFbetaRI SUMOylation cascade. Oncotarget. 2014;5(4):1071-82.

51.Chang CC, Lin DY, Fang HI, Chen RH,Shih HM. Daxx mediates the small ubiquitin-like modifier-dependent transcriptional repression of Smad4. J Biol Chem. 2005;280(11):10164-73.

52.Nait Achour T, Sentis S, Teyssier C, Philippat A, Lucas A, Corbo L, et al. Transcriptional repression of estrogen receptor alpha signaling by SENP2 in breast cancer cells. Mol Endocrinol. 2014;28(2):183-96.

53.Geng P, Zhang Y, Liu X, Zhang N, Liu Y, Liu X, et al. Automethylation of protein arginine methyltransferase 7 and its impact on breast cancer progression. The FASEB Journal. 2017;31(6):2287-2300.

54.Powers MA, Fay MM, Factor RE, Welm AL,Ullman KS. Protein arginine methyltransferase 5 accelerates tumor growth by arginine methylation of the tumor suppressor programmed cell death 4. Cancer Res. 2011;71(16):5579-87.

55.Yin S, Liu L, Brobbey C, Palanisamy V, Ball LE, Olsen SK, et al. PRMT5-mediated arginine methylation activates AKT kinase to govern tumorigenesis. Nat Commun. 2021;12(1):3444.

56.Jansson M, Durant ST, Cho EC, Sheahan S, Edelmann M, Kessler B, et al. Arginine methylation regulates the p53 response. Nat Cell Biol. 2008;10(12):1431-9.

57.Wang L, Zhao Z, Meyer MB, Saha S, Yu M, Guo A, et al. CARM1 methylates chromatin remodeling factor BAF155 to enhance tumor progression and metastasis. Cancer Cell. 2014;25(1):21-36.

58.Le Romancer M, Treilleux I, Leconte N, Robin-Lespinasse Y, Sentis S, Bouchekioua-Bouzaghou K, et al. Regulation of estrogen rapid signaling through arginine methylation by PRMT1. Mol Cell. 2008;31(2):212-21.

59.Yao R, Wang Y, Han D, Ma Y, Ma M, Zhao Y, et al. Lysines 207 and 325 methylation of WDR5 catalyzed by SETD6 promotes breast cancer cell proliferation and migration. Oncol Rep. 2018;40(5):3069-3077.

60.Huang J, Perez-Burgos L, Placek BJ, Sengupta R, Richter M, Dorsey JA, et al. Repression of p53 activity by Smyd2-mediated methylation. Nature. 2006;444(7119):629-32.

61.Hamamoto R, Saloura V,Nakamura Y. Critical roles of non-histone protein lysine methylation in human tumorigenesis. Nat Rev Cancer. 2015;15(2):110-24.

62.Zhang X, Tanaka K, Yan J, Li J, Peng D, Jiang Y, et al. Regulation of estrogen receptor alpha by histone methyltransferase SMYD2-mediated protein methylation. Proc Natl Acad Sci U S A. 2013;110(43):17284-9.

63.Chen JY, Li CF, Chu PY, Lai YS, Chen CH, Jiang SS, et al. Lysine demethylase 2A promotes stemness and angiogenesis of breast cancer by upregulating Jagged1. Oncotarget. 2016;7(19):27689-710.

64.Chen JY, Luo CW, Lai YS, Wu CC,Hung WC. Lysine demethylase KDM2A inhibits TET2 to promote DNA methylation and silencing of tumor suppressor genes in breast cancer. Oncogenesis. 2017;6(8):e369.

65.Poulard C, Rambaud J, Hussein N, Corbo L,Le Romancer M. JMJD6 regulates ERalpha methylation on arginine. PLoS One. 2014;9(2):e87982.

66.Zhu J, Zhao C, Kharman-Biz A, Zhuang T, Jonsson P, Liang N, et al. The atypical ubiquitin ligase RNF31 stabilizes estrogen receptor alpha and modulates estrogen-stimulated breast cancer cell proliferation. Oncogene. 2014;33(34):4340-51.

67.Zhu J, Zhuang T, Yang H, Li X, Liu H,Wang H. Atypical ubiquitin ligase RNF31: the nuclear factor modulator in breast cancer progression. BMC Cancer. 2016;16(1):

68.Haas TL, Emmerich CH, Gerlach B, Schmukle AC, Cordier SM, Rieser E, et al. Recruitment of the linear ubiquitin chain assembly complex stabilizes the TNF-R1 signaling complex and is required for TNF-mediated gene induction. Mol Cell. 2009;36(5):831-44.

69.Liu J, Zhang C, Zhao Y, Yue X, Wu H, Huang S, et al. Parkin targets HIF-1alpha for ubiquitination and degradation to inhibit breast tumor progression. Nat Commun. 2017;8(1):1823.

70.Zhang Y, Liao XH, Xie HY, Shao ZM,Li DQ. RBR-type E3 ubiquitin ligase RNF144A targets PARP1 for ubiquitin-dependent degradation and regulates PARP inhibitor sensitivity in breast cancer cells. Oncotarget. 2017;8(55):94505-94518.

71.Wu YH, Hong CW, Wang YC, Huang WJ, Yeh YL, Wang BJ, et al. A novel histone deacetylase inhibitor TMU-35435 enhances etoposide cytotoxicity through the proteasomal degradation of DNA-PKcs in triple-negative breast cancer. Cancer Lett. 2017;40079-88.

72.Harlen KM,Churchman LS. The code and beyond: transcription regulation by the RNA polymerase II carboxy-terminal domain. Nat Rev Mol Cell Biol. 2017;18(4):263-273.

73.Sharma P, Lioutas A, Fernandez-Fuentes N, Quilez J, Carbonell-Caballero J, Wright RHG, et al. Arginine Citrullination at the C-Terminal Domain Controls RNA Polymerase II Transcription. Mol Cell. 2019;73(1):84-96 e7.

74.Horibata S, Rogers KE, Sadegh D, Anguish LJ, McElwee JL, Shah P, et al. Role of peptidylarginine deiminase 2 (PAD2) in mammary carcinoma cell migration. BMC Cancer. 2017;17(1):378.

75.Stadler SC, Vincent CT, Fedorov VD, Patsialou A, Cherrington BD, Wakshlag JJ, et al. Dysregulation of PAD4-mediated citrullination of nuclear GSK3beta activates TGF-beta signaling and induces epithelial-to-mesenchymal transition in breast cancer cells. Proc Natl Acad Sci U S A. 2013;110(29):11851-6.

76.Hsu TH, Jiang SY, Chang WL, Eckert RL, Scharadin TM,Chang TC. Involvement of RARRES3 in the regulation of Wnt proteins acylation and signaling activities in human breast cancer cells. Cell Death Differ. 2015;22(9):1561.

77.Babina IS, McSherry EA, Donatello S, Hill AD,Hopkins AM. A novel mechanism of regulating breast cancer cell migration via palmitoylation-dependent alterations in the lipid raft affiliation of CD44. Breast Cancer Res. 2014;16(1):R19.

78.Gagnoux-Palacios L, Dans M, van't Hof W, Mariotti A, Pepe A, Meneguzzi G, et al. Compartmentalization of integrin alpha6beta4 signaling in lipid rafts. J Cell Biol. 2003;162(7):1189-96.

79.Mitchell DA, Vasudevan A, Linder ME,Deschenes RJ. Protein palmitoylation by a family of DHHC protein S-acyltransferases. J Lipid Res. 2006;47(6):1118-27.

80.Sharma C, Rabinovitz I,Hemler ME. Palmitoylation by DHHC3 is critical for the function, expression, and stability of integrin alpha6beta4. Cell Mol Life Sci. 2012;69(13):2233-44.

**Table S2.** **Inhibitors targeting PTM-associated enzymes to regulate breast cancer progression**

| **Type of PTMs** | **Agents** | **Target points** | **Target category** | **Function** | **Ref.** |
| --- | --- | --- | --- | --- | --- |
| Acetylation | Remodelin | KAT  (NAT10 inhibitor) | All Breast cancer  subtypes | Sensitive breast cancer cells to DNA-damaging chemotherapy and radiotherapy | [1] |
|  | TH1834 | KAT  (TIP60 inhibitor) | All Breast cancer  subtypes | Induce apoptosis and increase unrepaired DNA damage after DNA-damaging therapeutic | [2, 3] |
|  | ICG-001 | KAT  (CBP) | ER+ subtype | Block the β-catenin/CBP interaction, inhibit the EMT and invasion of MCF-7 cells | [4] |
|  | Panobinostat | KDAC  (pan-HDAC inhibitor) | All Breast cancer  subtypes | Induce endoplasmic reticulum and cell death | [5] |
|  | Vorinostat |  | All Breast cancer  subtypes | Induce cell cycle arrest and cell apoptosis | [6, 7] |
|  | PTACH |  | TNBC subtype | Exert potent growth inhibition in MDA-MB-231 cells | [8] |
|  | Dacinostat  (NVP-LAQ84) |  | All Breast cancer  subtypes | Inhibit cell proliferation by inducing cell cycle arrest and apoptosis | [9] |
|  | Valproic acid | KDAC  (class I and II HDAC inhibitor) | HER2+ subtype | Inhibit cell proliferation by inducing cell cycle arrest and apoptosis in HER2 positive breast cancer cells | [10] |
|  | Trichostatin A |  | TNBC subtype  HER2+ subtype | Promote the re-expression of ERα in ER-negative breast cancer cells, inhibit cell migration and invasion | [6, 7, 11-13] |
|  | CUDC-101 |  | TNBC subtype | Inhibit tumor growth in MDA-MB-468 breast cancer model | [14] |
|  | Tucidinostat  (Chidamide) |  | ER+ subtype | Inhibit MCF-7 cells growth both *in vivo* and *in vitro* | [15] |
|  | Quisinostat  (JNJ-26481585) | KDAC  (class I HDAC inhibitor) | All Breast cancer  subtypes | Show broad spectrum antiproliferation activity in breast cancer cell lines | [16] |
|  | Mocetinostat | KDAC  (class I and IV  HDAC inhibitor) | TNBC subtype | Inhibit the proliferation of MDA-MB 231cells | [17] |
|  | Entinostat | KDAC  (Class I HDAC inhibitor) | All Breast cancer  subtypes | Induce cell apoptosis by inhibit the expression of Bcl-2 | [18] |
|  | LMK-235 | KDAC  (selective HDAC5  Inhibitor) | All Breast cancer  subtypes | Inhibit cell growth and induce apoptosis | [19] |
|  | Tubacin | KDAC  (selective HDAC6  Inhibitor) | ER+ subtype | Inhibit estradiol-stimulated cell migration and invasion in MCF-7 cells | [11, 20] |
|  | Ricolinostat |  | All Breast cancer  subtypes | Inhibit cell migration and invasion | [21] |
|  | SKLB-23bb |  | HER2+ subtype | Show antiproliferation activity in SKBR3 cells | [22] |
|  | Citarinostat  (ACY241) |  | TNBC subtype  ER+ subtype | Increase the hyperacetylation of α-tubulin, significantly reduce proliferation and promote apoptosis in MDA-MB231 and T47D cells when combined with paclitaxel | [23] |
|  | Sirtinol | KDAC  (pan-sirtuin inhibitor) | HER2+ subtype | Increase the Sensitivity to Lapatinib | [24] |
|  | Salermide | KDAC  (selective sirtuin1and 2 inhibitor) | TNBC subtype  ER+ subtype | Increase the acetylation of p53 and induce the apoptosis of MDA-MB-231 and MCF-7 cells | [25] |
|  | Splitomicin | KDAC  (selective sirtuin1 inhibitor) | ER+ subtype | Decrease the colony formation ability in MCF-7 cells | [26] |
|  | Thiomyristoyl | KDAC  (selective sirtuin2 inhibitor) | All Breast cancer  subtypes | Exert antiproliferation in breast cell lines both *in vivo* and *in vitro* through promote c-myc ubiquitination | [27] |
|  | JQ1 | Bromodomain  (BET bromodomain  Inhibitor) | All Breast cancer  subtypes | Inhibit the tumorigenesis induced by the interaction between acetylated twist and BRD4 | [28, 29] |
| Glycosylation | Tunicamycin | Inhibit N-linked  glycosylation | All Breast cancer  subtypes | Induce the expression of p21 and p27 and reduce the expression of cyclin D1 and increase the sensitivity to chemotherapy | [30, 31] |
|  | 2-DG | Monosaccharide analog  (Glucose analog) | All Breast cancer  subtypes | Alter glycan structure and disturb the generation and elongation of oligosaccharide chains | [32] |
|  | 2-KetoGal | Monosaccharide analog  (GalNAc analog) | All Breast cancer  subtypes | Alter the structure of  O-GalNAc glycans | [33] |
|  | 6-Azidofucose | Monosaccharide analog  (Fucose analog) | All Breast cancer  subtypes | Alter glycan structure and disturb the generation and elongation of oligosaccharide chains | [34] |
|  | Ac4-5SGlcNAc | OGT | All Breast cancer  subtypes | Decrease global O-GlcNAc level | [35, 36] |
|  | OSMI-1 | OGT | TNBC subtype | Decrease global O-GlcNAc level, lead to cell death and growth inhibition in TNBC | [35-37] |
|  | Swainsonine | α-mannosidase | All Breast cancer  subtypes | Inhibit PD-L1 N-linked  glycosylation and promote cytotoxic T cell activity | [38, 39] |
|  | 2F-Peracetyl-Fucose | FUT | All Breast cancer  subtypes | Reduce the cells to adhere to E-selectin and inhibit the expression of growth factors | [40] |
|  | Ginsenoside Rg3 | FUT4 | TNBC subtype | Reduce the expression of FUT4 and inhibit fucosylation modification, induce apoptosis in MDA-MB 231cells | [41] |
| Sumoylation | Ginkgolic acids C15:1 | SUMO E1 activating enzymes | All Breast cancer  subtypes | Induce cell autophagy;  inhibit the activation of RAC1 induced cell migration and invasion | [42, 43] |
|  | 2-D08 | SUMO E2 conjugating enzyme | All Breast cancer  subtypes | Induce cell autophagy;  inhibit the activation of RAC1 induced cell migration and invasion | [43, 44] |
| Methylation | DCLX069 | PRMT  (PRMT1 inhibitor) | ER+ subtype | Occupy SAM binding site and repress MCF-7 cell proliferation | [45] |
|  | DCLX078 |  | ER+ subtype | Occupy SAM binding site and repress MCF-7 cell proliferation | [45] |
|  | C-7280948 |  | ER+ subtype | Show anticancer activity in MCF-7 cells | [46] |
|  | GSK591 | PRMT  (PRMT5 inhibitor) | All Breast cancer  subtypes | Inhibit breast CSCs proliferation and self-renewal | [47] |
|  | GSK3326595 | PRMT  (PRMT5 inhibitor) | All Breast cancer  subtypes | Inhibit the activation of AKT signal pathway | [48] |
|  | DC_C66 | PRMT  (CARM1inhibitor) | ER+ subtype | Inhibit the proliferation of MCF-7cells | [49] |
|  | DC_C11 | PRMT  (CARM1inhibitor) | ER+ subtype | Inhibit the proliferation of MCF-7cells | [49] |
|  | MS1943 | PKMT  (EZH2 inhibitor) | TNBC subtype | Inhibit the proliferation of multiple TNBC cells | [50] |
|  | NCGC00244536 | PKDM  (KDM4B inhibitor) | ER+ subtype | Inhibit the growth of MCF-7cells | [51] |
|  | PBIT | PKDM  (KDM5B inhibitor) | TNBC subtype  ER+ subtype | Inhibit the proliferation of UACC-812 and MCF-7cells | [52] |
|  | Daminozide | PKDM  (KDM2A inhibitor) | TNBC subtype | Inhibit CSCs and increase the sensitivity of cisplatin in MDA-MB-231 cells | [53, 54] |
| Ubiquitination | WP1130 | USP  (USP9X inhibitor) | All Breast cancer  subtypes | Promote chemotherapy induced tumor cell death | [55] |
|  | ML364 | USP  (USP2 inhibitor) | ER+ subtype | Induce cyclin D1 degradation and cause cell cycle arrest in MCF-7 cells | [56] |
|  | b-AP15 | USP  (USP14 inhibitor) | TNBC subtype | Inhibit tumor growth in 4T1 breast cancer model | [57] |
|  | Nutlin-3 | P53-MDM2 | All Breast cancer  subtypes | Inhibit MDM2- dependent P53 ubiquitin degradation and cause cell cycle G1 arrest | [58-60] |
|  | SP-141 | MDM2 | All Breast cancer  subtypes | Promote MDM2 auto-ubiquitination and degradation | [61] |
| Citrullination | Cl-Amidine | PADs inhibitor | All Breast cancer  subtypes | Inhibit the proliferation and migration of breast cancer cells | [62-64] |
|  | BB-Cl-Amidine | PADs inhibitor | All Breast cancer  subtypes | Inhibit the proliferation and migration of breast cancer cells | [62, 64] |
|  | D-Cl-amidine | PADs  (PAD1 inhibitor) | TNBC subtype | Decrease cell viability in MDA-MB-231 cells | [65] |
| Palmitoylation | Curcumin | PAT  (DHHC3 inhibitor) | All Breast cancer  subtypes | Inhibit the migration of breast cancer cells | [66] |

1.Liu HY, Liu YY, Yang F, Zhang L, Zhang FL, Hu X, et al. Acetylation of MORC2 by NAT10 regulates cell-cycle checkpoint control and resistance to DNA-damaging chemotherapy and radiotherapy in breast cancer. Nucleic Acids Res. 2020;48(7):3638-3656.

2.Gao C, Bourke E, Scobie M, Famme MA, Koolmeister T, Helleday T, et al. Rational design and validation of a Tip60 histone acetyltransferase inhibitor. Sci Rep. 2014;45372.

3.Guo P, Chen W, Li H, Li M,Li L. The Histone Acetylation Modifications of Breast Cancer and their Therapeutic Implications. Pathol Oncol Res. 2018;24(4):807-813.

4.Yan D, Avtanski D, Saxena NK,Sharma D. Leptin-induced epithelial-mesenchymal transition in breast cancer cells requires beta-catenin activation via Akt/GSK3- and MTA1/Wnt1 protein-dependent pathways. J Biol Chem. 2012;287(11):8598-612.

5.Rao R, Nalluri S, Kolhe R, Yang Y, Fiskus W, Chen J, et al. Treatment with panobinostat induces glucose-regulated protein 78 acetylation and endoplasmic reticulum stress in breast cancer cells. Mol Cancer Ther. 2010;9(4):942-52.

6.Huang L,Pardee AB. Suberoylanilide hydroxamic acid as a potential therapeutic agent for human breast cancer treatment. Mol Med. 2000;6(10):849-66.

7.Riaz SK, Saeed M,Malik M. Clinical and Therapeutic Implications of Histone Acetylation in Breast Cancer. West Indian Med J. 2015;65(2):337-344.

8.Suzuki T, Nagano Y, Kouketsu A, Matsuura A, Maruyama S, Kurotaki M, et al. Novel inhibitors of human histone deacetylases: design, synthesis, enzyme inhibition, and cancer cell growth inhibition of SAHA-based non-hydroxamates. J Med Chem. 2005;48(4):1019-32.

9.Atadja P, Gao L, Kwon P, Trogani N, Walker H, Hsu M, et al. Selective growth inhibition of tumor cells by a novel histone deacetylase inhibitor, NVP-LAQ824. Cancer Res. 2004;64(2):689-95.

10.Mawatari T, Ninomiya I, Inokuchi M, Harada S, Hayashi H, Oyama K, et al. Valproic acid inhibits proliferation of HER2-expressing breast cancer cells by inducing cell cycle arrest and apoptosis through Hsp70 acetylation. Int J Oncol. 2015;47(6):2073-81.

11.Rey M, Irondelle M, Waharte F, Lizarraga F,Chavrier P. HDAC6 is required for invadopodia activity and invasion by breast tumor cells. Eur J Cell Biol. 2011;90(2-3):128-35.

12.Yang X, Ferguson AT, Nass SJ, Phillips DL, Butash KA, Wang SM, et al. Transcriptional activation of estrogen receptor alpha in human breast cancer cells by histone deacetylase inhibition. Cancer Res. 2000;60(24):6890-4.

13.Vigushin DM, Ali S, Pace PE, Mirsaidi N, Ito K, Adcock I, et al. Trichostatin A is a histone deacetylase inhibitor with potent antitumor activity against breast cancer in vivo. Clin Cancer Res. 2001;7(4):971-6.

14.Cai X, Zhai HX, Wang J, Forrester J, Qu H, Yin L, et al. Discovery of 7-(4-(3-ethynylphenylamino)-7-methoxyquinazolin-6-yloxy)-N-hydroxyheptanamide (CUDc-101) as a potent multi-acting HDAC, EGFR, and HER2 inhibitor for the treatment of cancer. J Med Chem. 2010;53(5):2000-9.

15.Ning ZQ, Li ZB, Newman MJ, Shan S, Wang XH, Pan DS, et al. Chidamide (CS055/HBI-8000): a new histone deacetylase inhibitor of the benzamide class with antitumor activity and the ability to enhance immune cell-mediated tumor cell cytotoxicity. Cancer Chemother Pharmacol. 2012;69(4):901-9.

16.Arts J, King P, Marien A, Floren W, Belien A, Janssen L, et al. JNJ-26481585, a novel "second-generation" oral histone deacetylase inhibitor, shows broad-spectrum preclinical antitumoral activity. Clin Cancer Res. 2009;15(22):6841-51.

17.Fournel M, Bonfils C, Hou Y, Yan PT, Trachy-Bourget MC, Kalita A, et al. MGCD0103, a novel isotype-selective histone deacetylase inhibitor, has broad spectrum antitumor activity in vitro and in vivo. Mol Cancer Ther. 2008;7(4):759-68.

18.Raha P, Thomas S, Thurn KT, Park J,Munster PN. Combined histone deacetylase inhibition and tamoxifen induces apoptosis in tamoxifen-resistant breast cancer models, by reversing Bcl-2 overexpression. Breast Cancer Res. 2015;1726.

19.Li A, Liu Z, Li M, Zhou S, Xu Y, Xiao Y, et al. HDAC5, a potential therapeutic target and prognostic biomarker, promotes proliferation, invasion and migration in human breast cancer. Oncotarget. 2016;7(25):37966-37978.

20.Saji S, Kawakami M, Hayashi S, Yoshida N, Hirose M, Horiguchi S, et al. Significance of HDAC6 regulation via estrogen signaling for cell motility and prognosis in estrogen receptor-positive breast cancer. Oncogene. 2005;24(28):4531-9.

21.Sun Y, Sun J, Lungchukiet P, Quarni W, Yang S, Zhang X, et al. Fe65 Suppresses Breast Cancer Cell Migration and Invasion through Tip60 Mediated Cortactin Acetylation. Sci Rep. 2015;511529.

22.Yang Z, Wang T, Wang F, Niu T, Liu Z, Chen X, et al. Discovery of Selective Histone Deacetylase 6 Inhibitors Using the Quinazoline as the Cap for the Treatment of Cancer. J Med Chem. 2016;59(4):1455-70.

23.Huang P, Almeciga-Pinto I, Jarpe M, van Duzer JH, Mazitschek R, Yang M, et al. Selective HDAC inhibition by ACY-241 enhances the activity of paclitaxel in solid tumor models. Oncotarget. 2017;8(2):2694-2707.

24.Mahmud Z, Gomes AR, Lee HJ, Aimjongjun S, Jiramongkol Y, Yao S, et al. EP300 and SIRT1/6 Co-Regulate Lapatinib Sensitivity Via Modulating FOXO3-Acetylation and Activity in Breast Cancer. Cancers (Basel). 2019;11(8):

25.Lara E, Mai A, Calvanese V, Altucci L, Lopez-Nieva P, Martinez-Chantar ML, et al. Salermide, a Sirtuin inhibitor with a strong cancer-specific proapoptotic effect. Oncogene. 2009;28(6):781-91.

26.Ota H, Tokunaga E, Chang K, Hikasa M, Iijima K, Eto M, et al. Sirt1 inhibitor, Sirtinol, induces senescence-like growth arrest with attenuated Ras-MAPK signaling in human cancer cells. Oncogene. 2006;25(2):176-85.

27.Jing H, Hu J, He B, Negron Abril YL, Stupinski J, Weiser K, et al. A SIRT2-Selective Inhibitor Promotes c-Myc Oncoprotein Degradation and Exhibits Broad Anticancer Activity. Cancer Cell. 2016;29(3):297-310.

28.Filippakopoulos P, Qi J, Picaud S, Shen Y, Smith WB, Fedorov O, et al. Selective inhibition of BET bromodomains. Nature. 2010;468(7327):1067-73.

29.Shi J, Wang Y, Zeng L, Wu Y, Deng J, Zhang Q, et al. Disrupting the interaction of BRD4 with diacetylated Twist suppresses tumorigenesis in basal-like breast cancer. Cancer Cell. 2014;25(2):210-25.

30.Peiris D, Spector AF, Lomax-Browne H, Azimi T, Ramesh B, Loizidou M, et al. Cellular glycosylation affects Herceptin binding and sensitivity of breast cancer cells to doxorubicin and growth factors. Sci Rep. 2017;743006.

31.Wang X, Xiong W,Tang Y. Tunicamycin suppresses breast cancer cell growth and metastasis via regulation of the protein kinase B/nuclear factor-kappaB signaling pathway. Oncol Lett. 2018;15(4):4137-4142.

32.Berthe A, Zaffino M, Muller C, Foulquier F, Houdou M, Schulz C, et al. Protein N-glycosylation alteration and glycolysis inhibition both contribute to the antiproliferative action of 2-deoxyglucose in breast cancer cells. Breast Cancer Res Treat. 2018;171(3):581-591.

33.Hang HC,Bertozzi CR. Ketone isosteres of 2-N-acetamidosugars as substrates for metabolic cell surface engineering. J Am Chem Soc. 2001;123(6):1242-3.

34.Rabuka D, Hubbard SC, Laughlin ST, Argade SP,Bertozzi CR. A chemical reporter strategy to probe glycoprotein fucosylation. J Am Chem Soc. 2006;128(37):12078-9.

35.Gloster TM, Zandberg WF, Heinonen JE, Shen DL, Deng L,Vocadlo DJ. Hijacking a biosynthetic pathway yields a glycosyltransferase inhibitor within cells. Nat Chem Biol. 2011;7(3):174-81.

36.Ortiz-Meoz RF, Jiang J, Lazarus MB, Orman M, Janetzko J, Fan C, et al. A small molecule that inhibits OGT activity in cells. ACS Chem Biol. 2015;10(6):1392-7.

37.Barkovskaya A, Seip K, Hilmarsdottir B, Maelandsmo GM, Moestue SA,Itkonen HM. O-GlcNAc Transferase Inhibition Differentially Affects Breast Cancer Subtypes. Sci Rep. 2019;9(1):5670.

38.Galustian C, Foulds S, Dye JF,Guillou PJ. Swainsonine, a glycosylation inhibitor, enhances both lymphocyte efficacy and tumour susceptibility in LAK and NK cytotoxicity. Immunopharmacology. 1994;27(2):165-72.

39.Li CW, Lim SO, Chung EM, Kim YS, Park AH, Yao J, et al. Eradication of Triple-Negative Breast Cancer Cells by Targeting Glycosylated PD-L1. Cancer Cell. 2018;33(2):187-201 e10.

40.Tu CF, Wu MY, Lin YC, Kannagi R,Yang RB. FUT8 promotes breast cancer cell invasiveness by remodeling TGF-beta receptor core fucosylation. Breast Cancer Res. 2017;19(1):111.

41.Kim BM, Kim DH, Park JH, Na HK,Surh YJ. Ginsenoside Rg3 Induces Apoptosis of Human Breast Cancer (MDA-MB-231) Cells. J Cancer Prev. 2013;18(2):177-85.

42.Fukuda I, Ito A, Hirai G, Nishimura S, Kawasaki H, Saitoh H, et al. Ginkgolic acid inhibits protein SUMOylation by blocking formation of the E1-SUMO intermediate. Chem Biol. 2009;16(2):133-40.

43.Lorente M, Garcia-Casas A, Salvador N, Martinez-Lopez A, Gabicagogeascoa E, Velasco G, et al. Inhibiting SUMO1-mediated SUMOylation induces autophagy-mediated cancer cell death and reduces tumour cell invasion via RAC1. J Cell Sci. 2019;132(20):

44.Kim YS, Keyser SG,Schneekloth JS, Jr. Synthesis of 2',3',4'-trihydroxyflavone (2-D08), an inhibitor of protein sumoylation. Bioorg Med Chem Lett. 2014;24(4):1094-7.

45.Xie Y, Zhou R, Lian F, Liu Y, Chen L, Shi Z, et al. Virtual screening and biological evaluation of novel small molecular inhibitors against protein arginine methyltransferase 1 (PRMT1). Org Biomol Chem. 2014;12(47):9665-73.

46.Bissinger EM, Heinke R, Spannhoff A, Eberlin A, Metzger E, Cura V, et al. Acyl derivatives of p-aminosulfonamides and dapsone as new inhibitors of the arginine methyltransferase hPRMT1. Bioorg Med Chem. 2011;19(12):3717-31.

47.Chiang K, Zielinska AE, Shaaban AM, Sanchez-Bailon MP, Jarrold J, Clarke TL, et al. PRMT5 Is a Critical Regulator of Breast Cancer Stem Cell Function via Histone Methylation and FOXP1 Expression. Cell Rep. 2017;21(12):3498-3513.

48.Yin S, Liu L, Brobbey C, Palanisamy V, Ball LE, Olsen SK, et al. PRMT5-mediated arginine methylation activates AKT kinase to govern tumorigenesis. Nat Commun. 2021;12(1):3444.

49.Ye F, Zhang W, Lu W, Xie Y, Jiang H, Jin J, et al. Identification of Novel Inhibitors against Coactivator Associated Arginine Methyltransferase 1 Based on Virtual Screening and Biological Assays. Biomed Res Int. 2016;20167086390.

50.Ma A, Stratikopoulos E, Park KS, Wei J, Martin TC, Yang X, et al. Discovery of a first-in-class EZH2 selective degrader. Nat Chem Biol. 2020;16(2):214-222.

51.Duan L, Rai G, Roggero C, Zhang QJ, Wei Q, Ma SH, et al. KDM4/JMJD2 Histone Demethylase Inhibitors Block Prostate Tumor Growth by Suppressing the Expression of AR and BMYB-Regulated Genes. Chem Biol. 2015;22(9):1185-96.

52.Sayegh J, Cao J, Zou MR, Morales A, Blair LP, Norcia M, et al. Identification of small molecule inhibitors of Jumonji AT-rich interactive domain 1B (JARID1B) histone demethylase by a sensitive high throughput screen. J Biol Chem. 2013;288(13):9408-17.

53.Chen JY, Li CF, Chu PY, Lai YS, Chen CH, Jiang SS, et al. Lysine demethylase 2A promotes stemness and angiogenesis of breast cancer by upregulating Jagged1. Oncotarget. 2016;7(19):27689-710.

54.Rose NR, Woon EC, Tumber A, Walport LJ, Chowdhury R, Li XS, et al. Plant growth regulator daminozide is a selective inhibitor of human KDM2/7 histone demethylases. J Med Chem. 2012;55(14):6639-43.

55.Pal A,Donato NJ. Ubiquitin-specific proteases as therapeutic targets for the treatment of breast cancer. Breast Cancer Res. 2014;16(5):461.

56.Davis MI, Pragani R, Fox JT, Shen M, Parmar K, Gaudiano EF, et al. Small Molecule Inhibition of the Ubiquitin-specific Protease USP2 Accelerates cyclin D1 Degradation and Leads to Cell Cycle Arrest in Colorectal Cancer and Mantle Cell Lymphoma Models. J Biol Chem. 2016;291(47):24628-24640.

57.D'Arcy P, Brnjic S, Olofsson MH, Fryknas M, Lindsten K, De Cesare M, et al. Inhibition of proteasome deubiquitinating activity as a new cancer therapy. Nat Med. 2011;17(12):1636-40.

58.Knights CD, Liu Y, Appella E,Kulesz-Martin M. Defective p53 post-translational modification required for wild type p53 inactivation in malignant epithelial cells with mdm2 gene amplification. J Biol Chem. 2003;278(52):52890-900.

59.Vassilev LT, Vu BT, Graves B, Carvajal D, Podlaski F, Filipovic Z, et al. In vivo activation of the p53 pathway by small-molecule antagonists of MDM2. Science. 2004;303(5659):844-8.

60.Carvajal D, Tovar C, Yang H, Vu BT, Heimbrook DC,Vassilev LT. Activation of p53 by MDM2 antagonists can protect proliferating cells from mitotic inhibitors. Cancer Res. 2005;65(5):1918-24.

61.Wang W, Qin JJ, Voruganti S, Srivenugopal KS, Nag S, Patil S, et al. The pyrido[b]indole MDM2 inhibitor SP-141 exerts potent therapeutic effects in breast cancer models. Nat Commun. 2014;55086.

62.Horibata S, Rogers KE, Sadegh D, Anguish LJ, McElwee JL, Shah P, et al. Role of peptidylarginine deiminase 2 (PAD2) in mammary carcinoma cell migration. BMC Cancer. 2017;17(1):378.

63.Chumanevich AA, Causey CP, Knuckley BA, Jones JE, Poudyal D, Chumanevich AP, et al. Suppression of colitis in mice by Cl-amidine: a novel peptidylarginine deiminase inhibitor. Am J Physiol Gastrointest Liver Physiol. 2011;300(6):G929-38.

64.Knight JS, Subramanian V, O'Dell AA, Yalavarthi S, Zhao W, Smith CK, et al. Peptidylarginine deiminase inhibition disrupts NET formation and protects against kidney, skin and vascular disease in lupus-prone MRL/lpr mice. Ann Rheum Dis. 2015;74(12):2199-206.

65.Bicker KL, Anguish L, Chumanevich AA, Cameron MD, Cui X, Witalison E, et al. D-amino acid based protein arginine deiminase inhibitors: Synthesis, pharmacokinetics, and in cellulo efficacy. ACS Med Chem Lett. 2012;3(12):1081-1085.

66.Coleman DT, Soung YH, Surh YJ, Cardelli JA,Chung J. Curcumin Prevents Palmitoylation of Integrin beta4 in Breast Cancer Cells. PLoS One. 2015;10(5):e0125399.

**Table S3. The PTM-associated clinical trials without published articles in breast cancer patients**

| **Type of PTMs** | **Identifiers**  **/Ref** | **Title** | **Phase** | **Diseases** | **Intervention** | **Status** |
| --- | --- | --- | --- | --- | --- | --- |
| Acetylation | NCT00777049 | Study of Panobinostat Monotherapy in Women With HER2-negative Locally Recurrent or Metastatic Breast Cancer | II | Breast Cancer | Drug: Panobinostat | Completed |
|  | NCT00567879 | A Trial of Panobinostat and Trastuzumab for Adult Female Patients With HER2 Positive Metastatic Breast Cancer (MBC) Whose Disease Has Progressed on or After Trastuzumab | I/II | Breast Cancer | Drug: Panobinostat  Drug: Trastuzumab | Terminated |
|  | NCT00777335 | Study of Panobinostat Monotherapy in Women With v-ERB-B2 Avian Erythroblastic Leukemia Viral Oncogene Homolog 2 (HER2) Positive Locally Recurrent or Metastatic Breast Cancer | II | Breast Cancer | Drug: Panobinostat | Terminated |
|  | NCT00788931 | A Trial l of Panobinostat Given in Combination With Trastuzumab and Paclitaxel in Adult Female Patients With HER2 Positive Metastatic Breast Cancer | I | HER-2 Positive Breast Cancer  Metastatic Breast Cancer | Drug: Panobinostat  Drug: Trastuzumab | Completed |
|  | NCT00632489 | LBH589 in Combination With Capecitabine Plus/Minus (±) Lapatinib in Breast Cancer Patients | I | Breast Cancer | Drug: Panobinostat  Drug: Capecitabine  Drug: Lapatinib | Completed |
|  | NCT01194908 | Re-expression of ER in Triple Negative Breast Cancers | I/II | Breast Cancer | Drug: Decitabine  Drug: Panobinostat  Drug: Tamoxifen | Terminated |
|  | NCT00993642 | ERB-B4 After Treatment With HDAC Inhibitor in ER+ Tamoxifen Refractory Breast Cancer | I | Breast Cancer | Drug: Panobinostat | Withdrawn |
|  | NCT02890069 | A Study of PDR001 in Combination With LCL161, Everolimus or Panobinostat | I | Colorectal Cancer Non-small Cell Lung Carcinoma (Adenocarcinoma), Triple Negative Breast Cancer, Renal Cell Carcinoma | Drug:LCL161  Drug:Everolimus  Drug:Panobinostat  Drug: BM076  Drug: HDM201 | Active, not recruiting |
|  | NCT03878524 | Serial Measurements of Molecular and Architectural Responses to Therapy (SMMART) PRIME Trial | I | Chronic Myelogenous Leukemia,  Hodgkin Lymphoma,  Non-Hodgkin Lymphoma,  Breast cancer…… | Drug:Abemaciclib  Drug:Abiraterone  Drug: Afatinib  Drug:Olaparib  Drug:Oxaliplatin  Drug:Palbociclib  Drug: Panobinostat  …… | Recruiting |
|  | NCT03742245 | Olaparib in Combination With Vorinostat in Patients With Relapsed/Refractory and/or Metastatic Breast Cancer | I | Breast Cancer Metastatic Breast Cancer | Drug:Olaparib  Drug: Vorinostat | Recruiting |
|  | NCT00416130 | Clinical Trial of SAHA in Patients With Breast Cancer | I/II | Breast Cancer | Drug: Vorinostat | Unknown status |
|  | NCT00719875 | HDAC Inhibitor Vorinostat (SAHA) With Capecitabine (Xeloda) Using a New Weekly Dose Regimen for Advanced Breast Cancer | I | Advanced Breast Cancer | Drug: Vorinostat | Completed |
|  | NCT00574587 | Trial for Locally Advanced Breast Cancer Using Vorinostat Plus Chemotherapy | I/II | Breast Cancer | Drug: Vorinostat  Drug:Paclitaxel  Drug:TrastuzumabDrug:Doxorubicin  Drug:Cyclophosphamide | Completed |
|  | NCT01194427 | A Study of Vorinostat and Tamoxifen in Newly Diagnosed Breast Cancer | II | Breast Cancer | Drug: Vorinostat Drug: Tamoxifen | Terminated |
|  | NCT00132002 | Suberoylanilide Hydroxamic Acid in Treating Patients With Progressive Stage IV Breast Cancer | II | Male Breast Cancer  Recurrent Breast Cancer  Stage IV Breast Cancer | Drug: vorinostat | Terminated |
|  | NCT01695057 | Vorinostat Before Surgery in Treating Patients With Triple-Negative Breast Cancer | Not Applicable | Stage II Breast Cancer  Stage IIIA Breast Cancer  Triple-negative Breast Cancer | Drug: vorinostat | Withdrawn |
|  | NCT01118975 | GCC 0845:Vorinostat and Lapatinib in Advanced Solid Tumors and Advanced Breast Cancer to Evaluate Response and Biomarkers | I/II | Breast Cancer  Neoplasm Metastasis | Drug: Vorinostat  Drug: Lapatinib | Terminated |
|  | NCT00262834 | Vorinostat in Treating Women Who Are Undergoing Surgery For Newly Diagnosed Stage I -III Breast Cancer | II | Stage I -III Breast Cancer | Drug: vorinostat | Completed |
|  | NCT04190056 | Pembrolizumab and Tamoxifen With or Without Vorinostat for the Treatment of Estrogen Receptor Positive Breast Cancer | II | Stage IV Breast Cancer | Drug:Tamoxifen  Drug: Vorinostat | Active, not recruiting |
|  | NCT00368875 | Phase I-II Study of Vorinostat, Paclitaxel, and Bevacizumab in Metastatic Breast Cancer | I/II | Male Breast Cancer  Stage IIIB Breast Cancer  Stage IIIC Breast Cancer  Stage IV Breast Cancer | Drug: vorinostat  Drug: paclitaxel | Completed |
|  | NCT01084057 | Ixabepilone and Vorinostat in Treating Patients With Metastatic Breast Cancer | I | Male Breast Cancer  Recurrent Breast Cancer  Stage IV Breast Cancer | Drug:vorinostat  Drug: ixabepilone | Completed |
|  | NCT00788112 | Vorinostat in Treating Women With Ductal Carcinoma in Situ of the Breast | I | Breast Cancer | Drug: vorinostat | Completed |
|  | NCT00258349 | Vorinostat and Trastuzumab in Treating Patients With Metastatic or Locally Recurrent Breast Cancer | I/II | Male Breast Cancer  Recurrent Breast Cancer  Stage IIIB Breast Cancer  Stage IIIC Breast Cancer  Stage IV Breast Cancer | Drug: vorinostat  Drug: trastuzumab | Completed |
|  | NCT01720602 | Vorinostat in Treating Patients With Stage IV Breast Cancer Receiving Hormone Therapy | Not Applicable | Male Breast Cancer  Recurrent Breast Cancer  Stage IV Breast Cancer | Drug: vorinostat  Drug:anastrozole  Drug:letrozole  Drug: exemestane\| | Completed |
|  | NCT01153672 | Vorinostat in Treating Patients With Stage IV Breast Cancer Receiving Aromatase Inhibitor Therapy | Not Applicable | Male Breast Cancer  Recurrent Breast Cancer  Stage IV Breast Cancer | Drug: vorinostat | Completed |
|  | NCT00126451 | A Clinical Trial of Oral Suberoylanilide Hydroxamic Acid (SAHA) in Patients With Relapsed or Refractory Breast, Colorectal and Non-Small Cell Lung Cancer (0683-011) | II | Breast Cancer  Colorectal Cancer  Non-small-cell Lung Carcinoma | Drug: MK0683, Drug: vorinostat, | Terminated |
|  | NCT01249443 | Paclitaxel and Carboplatin in Treating Patients With Metastatic or Recurrent Solid Tumors and HIV Infection | I | HIV Infection  Recurrent Solid Tumors | Drug: vorinostat  Drug: carboplatin  Drug: paclitaxel | Terminated |
|  | NCT00045006 | Suberoylanilide Hydroxamic Acid in Treating Patients With Advanced Cancer | I | Cancer | Drug: vorinostat | Completed |
|  | NCT01007695 | Molecular Signature of Valproic Acid in Breast Cancer With Functional Imaging Assessment - a Pilot | I | Cancer | Drug: Valproic Acid | Terminated |
|  | NCT01010854 | Valproic Acid in Combination With FEC100 for Primary Therapy in Patients With Breast Cancer | II | Breast Cancer | Drug: VPA  Drug: FEC100 | Terminated |
|  | NCT01552434 | Bevacizumab and Temsirolimus Alone or in Combination With Valproic Acid or Cetuximab in Treating Patients With Advanced or Metastatic Malignancy or Other Benign Disease | I | Advanced Malignant Neoplasm | Drug: Temsirolimus  Drug: Valproic Acid | Unknown status |
|  | NCT01171924 | A Phase Ib Expansion Study Investigating the Safety, Efficacy, and Pharmacokinetics of Intravenous CUDC-101 in Subjects With Advanced Head and Neck, Gastric, Breast, Liver and Non-small Cell Lung Cancer Tumors | I | Head and Neck Cancer  Liver Cancer  Breast Cancer  Gastric Cancer  Non-Small Cell Lung Cancer | Drug: CUDC-101 | Completed |
|  | NCT04465097 | Neoadjuvant Tucidinostat and Exemestane in Early Breast Cancer | II | Breast Cancer | Drug: Tucidinostat  Drug: Exemestane  Drug: Ovarian function suppression | Recruiting |
|  | NCT04582955 | Neoadjuvant Treatment of Early Triple-negative Breast Cancer With Chidamide and Chemotherapy | Not Applicable | Triple-negative Breast Cancer | Drug: Chidamide in combination with chemotherapy | Recruiting |
|  | NCT04999540 | Tucidinostat and Fulvestrant in Hormone-receptor Positive Advanced Breast Cancer | II | Breast Cancer | Drug: Tucidinostat  Drug: Fulvestrant | Not yet recruiting |
|  | NCT04192903 | Chidamide Combined With Cisplatin for Relapsed or Metastatic Triple-negative Breast Cancer | II | Triple-negative Breast Cancer | Drug: Chidamide combined with Cisplatin | Not yet recruiting |
|  | NCT05047848 | Clinical Study of Chidamide Combined With Fulvestrant in the Treatment of Hormone Receptor-positive Advanced Breast Cancer | Not Applicable | Advanced Breast Cancer | Drug: Chidamide  Drug: Fulvestrant | Recruiting |
|  | NCT05085626 | Fluzoparib in Combination With Chidamide or Camrelizumab for HRD Positive HER2 Negative Advanced Breast Cancer | II | Advanced HER2 Negative Breast Carcinoma  HRD+Breast Cancer | Drug:fluzoparib  Drug: Chidamide  Drug: camrelizumab | Recruiting |
|  | NCT00511576 | Study to Evaluate Combination Treatment of MGCD0103 and Docetaxel (Taxotere®) for Subjects With Advanced Cancer Tumors | I | Breast Cancer  Lung Cancer  Pulmonary Cancer  Non-Small-Cell Lung Carcinoma  Prostate Cancer  Gastric Cancer  Stomach Cancer | Drug: MGCD0103 Drug: Docetaxel | Terminated |
|  | NCT03473639 | A Pilot Study of the Combination of Entinostat With Capecitabine in High Risk Breast Cancer After Neo-adjuvant | I | Breast Cancer  Metastatic Breast Cancer | Drug: Entinostat  Drug: Capecitabine | Recruiting |
|  | NCT02820961 | Drug-Drug Interaction Study of Entinostat and Exemestane in Postmenopausal Women With ER+ Breast Cancer | I | Breast Cancer  Estrogen Receptor Positive Breast Cancer | Drug: Entinostat  Drug: Exemestane | Completed |
|  | NCT02708680 | Randomized Phase 2 Study of Atezolizumab and Entinostat in Patients With aTN Breast Cancer With Phase 1b Lead In | I/II | Breast Cancer | Drug: entinostat  Drug: atezolizumab | Unknown status |
|  | NCT03538171 | Ph3 Study of Exemestane With or Without Entinostat in Chinese Patients With Hormone Receptor-Positive, Locally Advanced or Metastatic Breast Cancer | III | Advanced Breast Cancer | Drug: Entinostat  Drug: Exemestane | Active, not recruiting |
|  | NCT02115594 | Phase 2 Study of Fulvestrant With and Without Entinostat in Postmenopausal Women With ER+ Advanced Breast | II | Breast Cancer | Drug: Fulvestrant  Drug: Entinostat | Withdrawn |
|  | NCT03361800 | Window of Opportunity Trial of Entinostat in Patients With Newly Diagnosed Stage I-IIIC,TNBC | I | Breast Cancer  Invasive Breast Cancer  TNBC | Drug: Entinostat | Terminated |
|  | NCT00828854 | A Phase 2, Multicenter Study of the Effect of the Addition of SNDX-275 to Continued Aromatase Inhibitor (AI) Therapy in Postmenopausal Women With ER+ Breast Cancer Whose Disease is Progressing | II | ER+ Breast Cancer | Drug: entinostat | Completed |
|  | NCT03291886 | Phase 2 Study of KHK2375 in Subjects With Advanced or Recurrent Breast Cancer | II | Advanced or Recurrent Breast Cancer | Drug: Entinostat  Drug: Exemestane | Active, not recruiting |
|  | NCT01434303 | Entinostat, Lapatinib Ditosylate and Trastuzumab in Treating Patients With Locally Recurrent or Distant Relapsed Metastatic Breast Cancer Previously Treated With Trastuzumab Only | I | HER2 Positive  Invasive Breast Carcinoma  Recurrent Breast Carcinoma  Stage IV Breast Cancer | Drug: Entinostat  Drug: Lapatinib  Drug: Trastuzumab | Completed |
|  | NCT01594398 | Study to Assess Food Effect on Pharmacokinetics of Entinostat in Subjects With Breast Cancer or Non-Small Cell Lung Cancer | I | Lung Cancer  Non- Small Cell Lung Cancer (NSCLC)  Breast Cancer  Estrogen Receptor Breast Cancer | Drug: entinostat\|Drug: Erlotinib\|Drug: Exemestane | Completed |
|  | NCT01234532 | Entinostat and Anastrozole in Treating Postmenopausal Women With TNBC That Can Be Removed by Surgery | II | Stage I Breast Cancer  Stage II Breast Cancer  Stage IIIA Breast Cancer  Triple-negative Breast Cancer | Drug: entinostat  Drug: anastrozole | Terminated |
|  | NCT02453620 | Entinostat, Nivolumab, and Ipilimumab in Treating Patients With Solid Tumors That Are Metastatic or Cannot Be Removed by Surgery or Locally Advanced or Metastatic HER2-Negative Breast | I | Breast Adenocarcinoma  Invasive Breast Carcinoma  Metastatic Breast Carcinoma  Metastatic Malignant Solid Neoplasm  Stage III Breast Cancer  Stage IV Breast Cancer Unresectable Solid Neoplasm | Drug: Entinostat  Biological: Ipilimumab  Biological: Nivolumab | Active, not recruiting |
|  | NCT00754312 | A Phase I, Multicenter, Open Label Study on the Effects of SNDX-275 on Expression of Biomarkers in Subjects With Newly Diagnosed Breast Cancer | I | Breast Cancer | Drug: SNDX-275 | Withdrawn |
|  | NCT03280563 | A Study of Multiple Immunotherapy-Based Treatment Combinations in Hormone Receptor (HR)-Positive Human Epidermal Growth Factor Receptor 2 (HER2)-Negative Breast Cancer | I/II | Breast Neoplasms | Drug: Atezolizumab (MPDL3280A), an engineered anti-programmed death-ligand 1 (PD-L1) antibody  Drug: Bevacizumab  Drug: Entinostat  Drug: Exemestane  Drug: Fulvestrant  Drug: Ipatasertib  Drug: Tamoxifen  Drug: Abemaciclib | Recruiting |
|  | NCT02897778 | Cardiac Safety Study of Entinostat in Men and Women With Advanced Solid Tumors | I | Neoplasms, Glandular and Epithelial  Bronchial Neoplasms  Lung Neoplasms  Respiratory Tract Neoplasms  Thoracic Neoplasms  Digestive System Neoplasms  Endocrine Gland Neoplasms  Carcinoma,  Breast Diseases  Renal Neoplasm  Solid Tumors | Drug: Entinostat | Completed |
|  | NCT02909452 | Continuation Study of Entinostat in Combination With Pembrolizumab in Patients With Advanced Solid Tumors | I | Neoplasms, Glandular and Epithelial  Bronchial Neoplasms  Lung Neoplasms  Respiratory Tract Neoplasms  Thoracic Neoplasms  Digestive System Neoplasms  Endocrine Gland Neoplasms  Carcinoma,  Breast Diseases  Renal Neoplasm  Solid Tumors | Drug: Entinostat  Drug:  Pembrolizumab | Unknown status |
|  | NCT00020579 | MS-275 in Treating Patients With Advanced Solid Tumors or Lymphoma | I | Cancer | Drug: entinostat | Completed |
|  | NCT02632071 | ACY-1215 + Nab-paclitaxel in Metastatic Breast Cancer | I | Metastatic Breast Cancer\|Breast Carcinoma | Drug: Ricolinostat  Drug:Nab-paclitaxel | Completed |
| Methylation | NCT04676516 | A Phase II Window of Opportunity Trial of PRMT5 Inhibitor, GSK3326595, in Early Stage Breast Cancer | II | Breast Cancer | Drug: GSK3326595 | Not yet recruiting |
| Palmitoylation | NCT03980509 | A "Window Trial" on Curcumin for Invasive Breast Cancer Primary Tumors | I | Breast Cancer | Drug: Curcumin | Recruiting |
|  | NCT03847623 | Effect of Preoperative Curcumin in Breast Cancer Patients | Not Applicable | Breast Cancer | Dietary Supplement: Curcumin | Active, not recruiting |
|  | NCT03072992 | "Curcumin" in Combination With Chemotherapy in Advanced Breast Cancer | II | Advanced Breast Cancer  Metastatic Breast Cancer | Drug: Curcumin  Drug: Paclitaxel | Completed |
|  | NCT01740323 | Phase II Study of Curcumin vs Placebo for Chemotherapy-Treated Breast Cancer Patients Undergoing | II | Breast Cancer | Drug: Curcumin | Completed |
|  | NCT00852332 | Docetaxel With or Without a Phytochemical in Treating Patients With Breast Cancer | II | Breast Cancer | Dietary Supplement: Curcumin  Drug: Taxotere | Terminated |


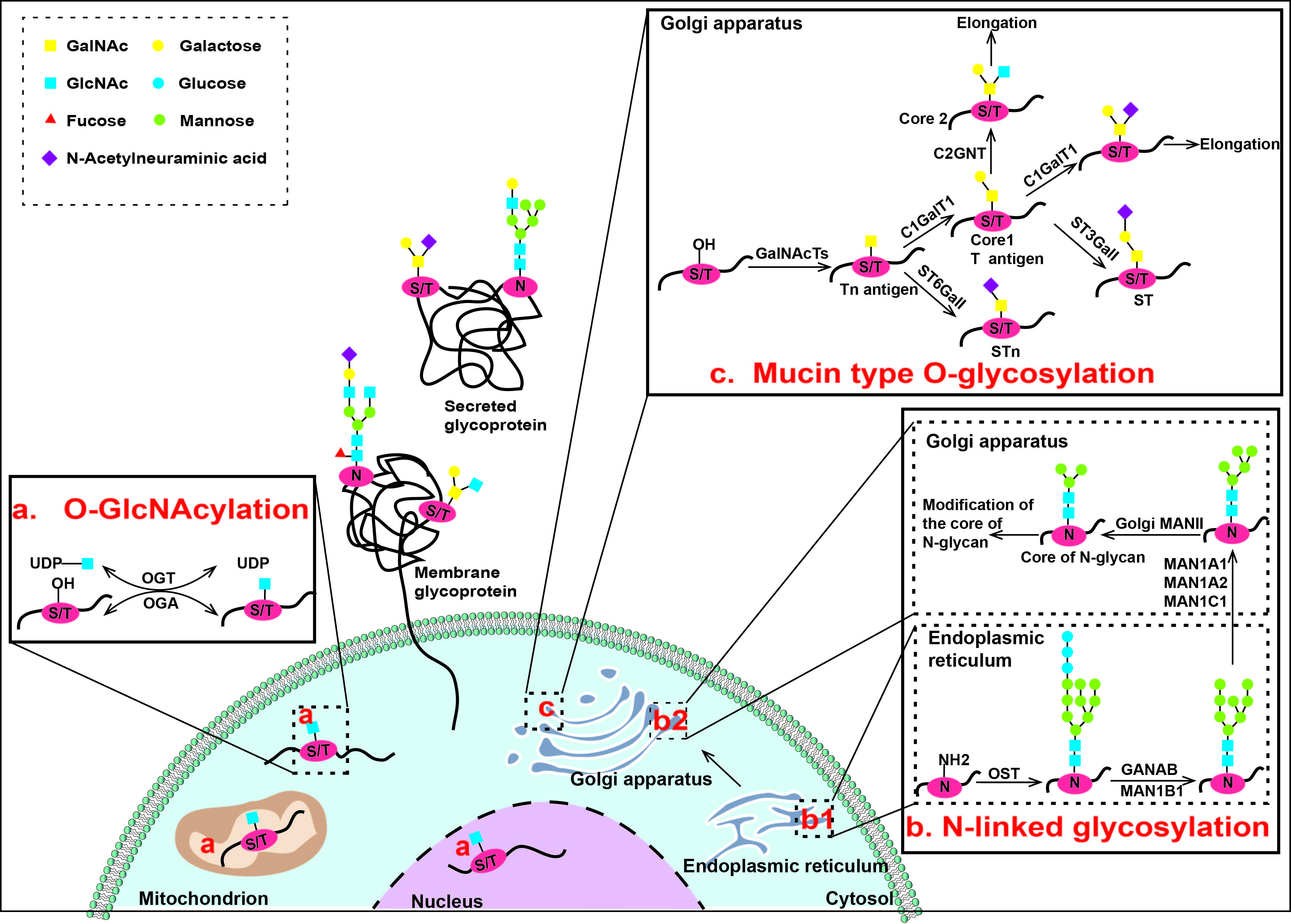


**Fig. S1 Different glycosylation modification in breast cancer. a.** Regulation of O-GlcNAcylation. **b.** Regulation of N-linked glycosylation. **c.** Regulation of mucin type O-glycosylation. S: serine; T: threonine; GalNAc-Tases: N-acetylgalactosaminyl transferases; STn: sialylated Tn; ST6GalI: β-galactosidase α 2,6-sialyltransferase I; C1GalT1: core 1 synthase glycoprotein-N-acetylgalactosamine 3-β-galactosyltransferase 1; ST3GalI: β-galactosidase α2,3-sialyltransferase I; N: asparagine; OST: oligosaccharyltransferase; MAN1B1: α-1,2-mannosidase I.


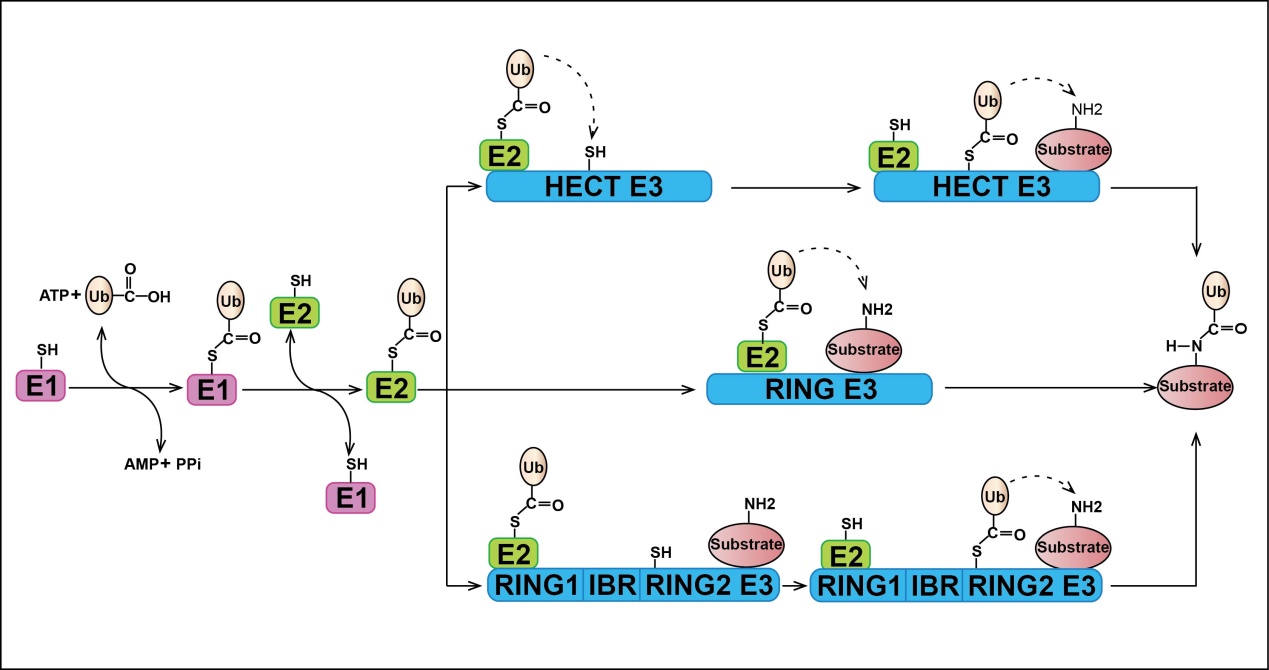


**Fig. S2 The mechanisms underlying protein ubiquitination.**

**
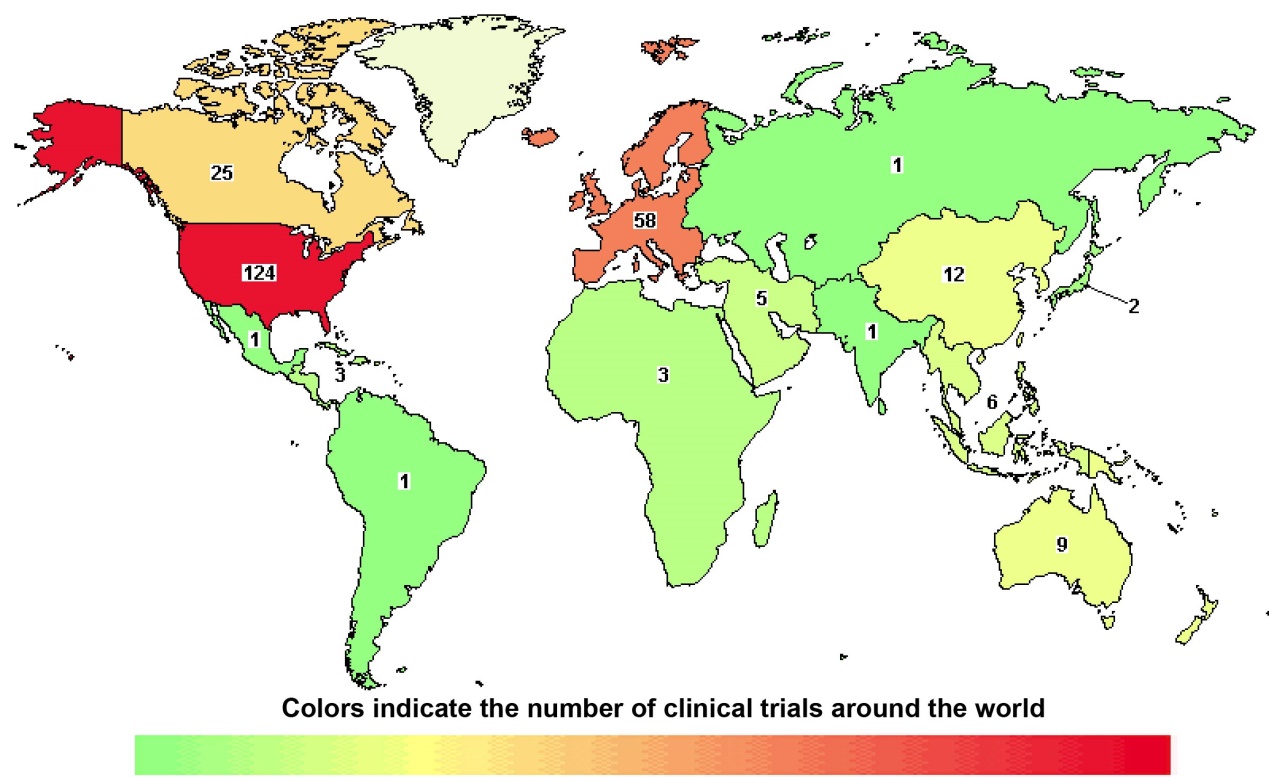
**

**Fig. S3 The distribution of protein PTM-associated clinical trials worldwide.**
